# A Phosphorylation Switch on Lon Protease Regulates Bacterial Type III Secretion System in Host

**DOI:** 10.1128/mBio.02146-17

**Published:** 2018-01-23

**Authors:** Xiaofeng Zhou, Doron Teper, Maxuel O. Andrade, Tong Zhang, Sixue Chen, Wen-Yuan Song, Nian Wang

**Affiliations:** a Department of Microbiology and Cell Science, Citrus Research and Education Center, Institute of Food and Agricultural Sciences, University of Florida, Lake Alfred, Florida, USA; b Department of Biology and University of Florida Genetics Institute, University of Florida, Gainesville, Florida, USA; c Department of Plant Pathology, University of Florida, Gainesville, Florida, USA; University of Massachusetts Amherst; Mass General Hospital

**Keywords:** Lon protease, type III secretion system, *Xanthomonas*

## Abstract

Most pathogenic bacteria deliver virulence factors into host cytosol through type III secretion systems (T3SS) to perturb host immune responses. The expression of T3SS is often repressed in rich medium but is specifically induced in the host environment. The molecular mechanisms underlying host-specific induction of T3SS expression is not completely understood. Here we demonstrate in *Xanthomonas citri* that host-induced phosphorylation of the ATP-dependent protease Lon stabilizes HrpG, the master regulator of T3SS, conferring bacterial virulence. Ser/Thr/Tyr phosphoproteome analysis revealed that phosphorylation of Lon at serine 654 occurs in the citrus host. In rich medium, Lon represses T3SS by degradation of HrpG via recognition of its N terminus. Genetic and biochemical data indicate that phosphorylation at serine 654 deactivates Lon proteolytic activity and attenuates HrpG proteolysis. Substitution of alanine for Lon serine 654 resulted in repression of T3SS gene expression in the citrus host through robust degradation of HrpG and reduced bacterial virulence. Our work reveals a novel mechanism for distinct regulation of bacterial T3SS in different environments. Additionally, our data provide new insight into the role of protein posttranslational modification in the regulation of bacterial virulence.

## IMPORTANCE

Type III secretion systems (T3SS) are an essential virulence trait of many bacterial pathogens because of their indispensable role in the delivery of virulence factors. However, expression of T3SS in the noninfection stage is energy consuming. Here, we established a model to explain the differential regulation of T3SS in host and nonhost environments. When *Xanthomonas* cells are grown in rich medium, the T3SS regulator HrpG is targeted by Lon protease for proteolysis. The degradation of HrpG leads to downregulated expression of HrpX and the *hrp*/*hrc* genes. When *Xanthomonas* cells infect the host, specific plant stimuli can be perceived and induce Lon phosphorylation at serine 654. Phosphorylation on Lon attenuates its proteolytic activity and protects HrpG from degradation. Consequently, enhanced stability of HrpG activates HrpX and turns on bacterial T3SS in the host. Our work provides a novel molecular mechanism underlying host-dependent activation of bacterial T3SS.

## INTRODUCTION

Bacterial pathogens are commonly present in both host and nonhost environments. In typical nonhost environments, bacteria face many stressors, including UV light, humidity, temperature, and drought (1, 2). Upon entering the host, bacterial pathogens are subjected to immune responses such as pathogen-associated molecular pattern-triggered immunity and effector-triggered immunity (3). To conquer host immune responses, bacterial cells can deliver type III effector (T3E) proteins, which are encoded by *hrp* (hypersensitive response [HR] and pathogenicity) and *hrc* (HR and conserved) gene clusters in plant-pathogenic bacteria, into host cytosol through type III secretion systems (T3SS) (3–5). Regulation of T3SS in *Xanthomonas* spp., a class of gammaproteobacteria causing plant diseases, depends on two master regulators, HrpG and HrpX, which are OmpR- and AraC-type transcriptional regulators, respectively (6–8). HrpG retains an extremely low expression level in rich medium; however, significant induction can be observed during bacterial infection of a host (9). Upregulation of HrpG activates the transcriptional activator HrpX, which binds to a conserved plant-inducible promoter box (TTCGC-N_15_-TTCGC) present in the promoter regions of most of *hrp*/*hrc* and T3E-encoding genes, activating T3SS expression (7, 8, 10).

Expression of the T3SS and effector genes is known to be suppressed in nonhost environments and induced in the host environment (11). Understanding the underlying induction mechanism of important virulence traits, including the T3SS, not only has broad implications in the study of plant-pathogen interactions and the molecular ecology of bacterial pathogens but also provides insights into disease control. In plant-pathogenic bacteria, T3SS gene expression is induced by plant-specific signals such as plant phenolic compounds and sugars and suitable physiological and chemical conditions, including low pH, low osmolarity, and low temperature (12–15). However, how T3SS expression is induced by host signals through an HrpG/X-dependent manner remains unknown.

Citrus canker is an economically significant disease caused by *Xanthomonas citri* subsp. *citri* (16). The disease is manifested in the appearance of pustules on fruits and leaves that become necrotic in the later stages. At a cellular level, canker disease symptoms are characterized by active manipulation of cell identity by the bacteria, causing hypertrophy and hyperplasia of the cells at the infection site (16). This manipulation of cell identity is mediated by the bacterial transcription activator-like T3E protein PthA4. PthA4 is translocated into the cell via the T3SS and directly activates the transcription of the citrus gene *CsLOB1*, which in turn promotes the alteration of cell identity (17).

Proteolysis enables virulence factors to be present in a short time window of the infectious process. One of the main proteolytic players is Lon protease, which is highly conserved and widely distributed in all domains of life. Lon protease consists of three domains: an N domain, an AAA+ module, and a proteolytic domain (P domain) (18). The N domain of Lon plays multiple roles in substrate binding, allosteric regulation of protease activity, and suppression of proteotoxic stress (19); the AAA+ module encodes a functional ATPase domain that provides energy for Lon-mediated protein digestion via ATP hydrolysis (20); and the P domain, containing a Ser-Lys dyad activation site, along with the AAA+ domain, forms a hexamer ring structure with an internal sequestered degradation chamber for proteolysis (21, 22). Substrate recognition by Lon protease depends on its N- or C-terminal amino acid sequence (also known as a degradation tag or degron). It is known that Lon regulates T3SS in many bacterial pathogens by degrading key regulatory proteins (16–18, 23, 24, 51). In the plant-pathogenic bacterium *Pseudomonas syringae*, the T3SS is predominantly controlled by an alternative sigma factor, HrpL. The transcription of HrpL is regulated by two enhancer binding proteins, HrpR and HrpS. It has been shown that Lon negatively regulates the T3SS by degrading HrpR in non-*hrp*-inducing medium (24).

In this study, we characterized the role of the Lon protease in T3SS regulation in *X. citri* subsp. *citri*. We found that Lon transcriptionally controls *hrp*/*hrc* gene expression by degrading the master regulator HrpG, thereby negatively regulating *X. citri* subsp. *citri* virulence and a nonhost HR. Interestingly, qualitative phosphoproteomics revealed that Lon can be phosphorylated at serine 654 in the host. Phosphorylated serine within the P domain impairs Lon proteolytic activity, leading to increased stability of HrpG. Our study uncovers an important mechanism by which bacterial pathogens efficiently adapt to host and nonhost environments. We also hypothesize that Lon phosphorylation could be a molecular switch that controls T3SS expression in bacterial pathogens in host and non-T3SS-inducing environments.

## RESULTS

### Phosphoproteome analysis reveals host-induced Lon protease phosphorylation at serine 654.

**FIG 1:**
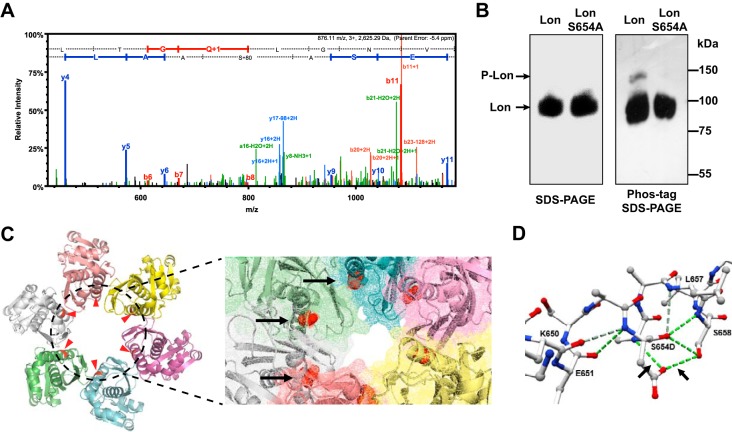
Phosphorylation of *X. citri* subsp. *citri* Lon at serine 654. (A) MS/MS spectrum assigned to LAApSASE of *X. citri* subsp. *citri* Lon protease. The b and y ions are red and blue, respectively. (B) Phos tag gel analysis of Lon. Lon and LonS654A were immunoprecipitated from an overnight *X. citri* subsp. *citri* culture with an anti-Lon antibody, separated by 10% SDS-PAGE without (left) or with 50 µM Phos tag (right) and 100 µM MnCl_2_, and immunoblotted with an anti-Lon antibody. (C) Protein model of the *X. citri* subsp. *citri* Lon protease P domain. The hexamer ring structure of the *X. citri* subsp. *citri* Lon P domain was built on the basis of the structure of the *E. coli* Lon P domain. The phosphorylation sites are highlighted in red and indicated by arrows. An enlarged image of the *X. citri* subsp. *citri* Lon P domain in the surface mode shows the degradation chamber with red highlighting phosphorylation sites. (D) Mutation analysis of serine 654 (S654) with aspartic acid (D) illustrated by a ball-and-stick model of the second α helix in the Lon P domain. H bonds are indicated by green dashed lines.

Protein phosphorylation on serine, threonine, and tyrosine residues is one of the common posttranslational modifications that are known mechanisms that regulate multiple cellular processes, including bacterial signaling and pathogenicity (25). To identify global phosphorylation events in *X. citri* subsp. *citri* and explore their roles in the regulation of bacterial virulence during host infection, comparative phosphoproteome analyses of *X. citri* subsp. *citri* were performed with cells grown in rich medium (NB [nutrient broth] medium, pH 7.0) and recovered from Duncan grapefruit plants at 5 days postinoculation. We successfully identified 53 phosphoproteins in rich medium versus 40 in plants, of which 7 were phosphorylated under both conditions (see Table S1 in the supplemental material). Among 53 proteins phosphorylated in rich medium, the percentages of p-Ser, p-Thr, and p-Tyr residues were 44.9, 46.9, and 8.2%, respectively (Fig. S1). Compared to the rich medium condition, the p-Ser and p-Thr residue percentages under the plant condition were increased to 50.0 and 47.4%, respectively, and the p-Tyr percentage was reduced to 2.6% (Fig. S1). In addition, phosphoproteins belonging to the Clusters of Orthologous Groups (COG) categories posttranslational modification, protein turnover, and chaperones were significantly enriched (Fisher’s exact test, *P* < 0.05) (Table S2). This indicates that protein phosphorylation may be tightly associated with protein posttranslational modification and quality control. Among such categories, an ATP-dependent Lon protease was found to be phosphorylated at serine 654 in the host but not in rich medium (Fig. 1A). Lon has been reported to be associated with the regulation of virulence in multiple pathogenic bacteria (26–28). The host-induced phosphorylation of Lon raises an intriguing possibility that proteolysis plays a distinct role in the regulation of virulence traits in the host.

10.1128/mBio.02146-17.1 FIG S1  Ratios of three types of phosphorylated residues. Each stacked bar represents the corresponding experimental condition indicated. Percentages of serine, threonine, and tyrosine phosphorylation under the corresponding conditions are shown. Download FIG S1, JPG file, 0.5 MB. Copyright © 2018 Zhou et al. 2018 Zhou et al. This content is distributed under the terms of the Creative Commons Attribution 4.0 International license.

10.1128/mBio.02146-17.4 TABLE S1  Phosphopeptides identified in *X. citri* subsp. *citri*. Download TABLE S1, DOCX file, 0.02 MB. Copyright © 2018 Zhou et al. 2018 Zhou et al. This content is distributed under the terms of the Creative Commons Attribution 4.0 International license.

10.1128/mBio.02146-17.5 TABLE S2  Comparison of COG assignments with regard to phosphoproteins identified under different experimental conditions in *X. citri* subsp. *citri*. Download TABLE S2, DOCX file, 0.02 MB. Copyright © 2018 Zhou et al. 2018 Zhou et al. This content is distributed under the terms of the Creative Commons Attribution 4.0 International license.

Serine 654 of *X. citri* subsp. *citri* Lon is located in the middle of the second α helix and is conserved in all *Xanthomonas* spp. The crystal structure of the *Escherichia coli* Lon P domain has been solved at 1.75 Å resolution (29). It is 70% identical to *X. citri* subsp. *citri* Lon (Fig. S2), suggesting that Lon employs a similar mechanism to degrade its protein substrates in *X. citri* subsp. *citri*. To further verify that serine 654 in *X. citri* subsp. *citri* Lon can be phosphorylated, an alanine substitution (S654A) was created and introduced into a *lon* mutant. Wild-type Lon and LonS654A were isolated from bacterial cultures grown in XVM2 medium (52) at pH 6.3 (mimicking the plant environment) by immunoprecipitation, and the phosphorylation state of Lon was monitored by immunoblot analysis with standard or Phos tag SDS-PAGE gels (30). When samples were separated on Phos tag gel, immunoblotting of wild-type Lon produced a second higher band representing the phosphorylated Lon in addition to that observed on a standard gel, which was not observed in the LonS654A variant (Fig. 1B).

10.1128/mBio.02146-17.2 FIG S2  Lon protease sequence alignment. The filled arrowhead indicates the phosphorylation site, and the hollow arrowhead indicates the Ser-Lys dyad activation site. Bsu, *Bacillus subtilis* subsp. *subtilis* 168 (NP_390699); Xac, *X. citri* subsp. *citri* (AAM35958); Eco, *E. coli* K-12 MG1655 (NP_414973); Pst, *Pseudomonas syringae* pv. *tomato* DC3000 (NP_793498). Download FIG S2, JPG file, 2 MB. Copyright © 2018 Zhou et al. 2018 Zhou et al. This content is distributed under the terms of the Creative Commons Attribution 4.0 International license.

We built a homology model of the *X. citri* subsp. *citri* Lon P domain by using the *E. coli* Lon structure as a template (Fig. 1C). The bottom view of the hexamer, with the highlighted phosphorylation sites, shows that the second α helix localizes at the inner edge of the center channel. In addition, an enlarged image in the surface mode clearly shows that the phosphorylated residues are exposed to the surface of the degradation channel. This indicates that phosphorylation at serine 654 may trigger a subtle conformational change in the Lon P domain, thus interfering with protease activity (Fig. 1C). Structural simulation using a phosphomimetic aspartic acid at position 654 of the P domain revealed the formation of two new H bonds between two structurally adjacent residues, A655 and S658, as shown in Fig. 1D.

### Lon protease negatively regulates bacterial virulence by repressing *hrc/hrp* gene transcription.

**FIG 2:**
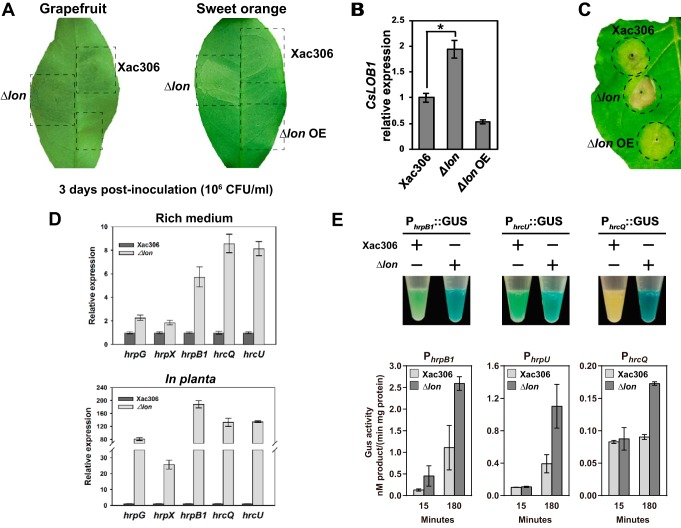
Lon negatively regulates bacterial virulence. (A) Grapefruit and sweet orange leaves were inoculated with wild-type strain 306 (Xac306), the Δ*lon* mutant, and a Lon-overexpressing strain (Δ*lon* OE) with a needleless syringe. The bacterial titer of the inoculum was adjusted to 10^6^ CFU ml^−1^. The canker symptoms induced by the corresponding strains were photographed at 3 days postinoculation. The experiments were repeated three times with comparable results, and only one representative leaf is shown. (B) *CsLOB1* expression levels in grapefruit leaves inoculated with wild-type strain 306, the Δ*lon* mutant, and the Δ*lon* OE strain. *CsLOB1* expression was monitored by qRT-PCR at 48 h postinoculation. The mean values ± the standard deviations (*n* = 3) are plotted. The asterisk indicates a statistically significant difference (*P* < 0.01, Student *t* test). (C) Fully expanded 4-week-old *N. benthamiana* leaves were inoculated with wild-type strain 306, the Δ*lon* mutant, and the Δ*lon* OE strain at a concentration of 10^8^ CFU ml^−1^. HRs were observed and photographed at 1 week postinoculation. The experiments were repeated three times with comparable results, and only one representative leaf is shown. (D) Comparison of relative *hrp*/*hrc* gene expression in wild-type strain 306 and the Δ*lon* mutant in rich medium (top) and *in planta* (bottom). RNA samples were extracted from bacterial cells either grown in NB medium to stationary phase or recovered from plant leaves at 4 days postinoculation. Relative *hrpG*, *hrpX*, *hrcQ*, *hrcU*, and *hrpB1* expression was monitored by qRT-PCR. The mean values ± the standard deviations (*n* = 3) are plotted. (E) The *hrpB1*, *hrcU*, and *hrcQ* promoters were fused with a GUS reporter gene, and the derivative plasmids were transferred into the wild type and the Δ*lon* mutant. Transformants were grown in rich medium overnight and stained with X-Gluc for 10 min. The experiments were repeated three times with comparable results, and only one tube is presented. Quantification of GUS activity was performed with *p*-nitrophenyl-β-d-glucuronide as the substrate. The mean values ± the standard deviations (*n* = 3) are plotted.

To determine the function of Lon protease in bacterial virulence, grapefruit and sweet orange plants were inoculated with the *lon* deletion mutant and Lon-overexpressing strains and the plants were monitored for canker symptoms. Lon protein expression was verified by immunoblotting with an anti-FLAG antibody (Fig. S3A). Deletion of *lon* induced pustule formation at the site of infection that was faster and stronger than that caused by wild-type *X. citri* subsp. *citri*. Pustule formation was inhibited by Lon overexpression, indicating that Lon negatively regulates *X. citri* subsp. *citri* virulence (Fig. 2A). The formation of canker pustules is tightly associated with the expression level of a citrus canker susceptibility gene, *CsLOB1*, which is activated by a T3E, PthA4 (17). We found that the expression of *CsLOB1* was significantly higher in *lon* mutant-inoculated leaves than in wild-type-inoculated leaves. On the contrary, leaves inoculated with the strain overexpressing Lon showed a significantly lower level of *CsLOB1* expression than leaves inoculated with the wild type (Fig. 2B). In addition, the *lon* mutant induced an earlier HR in a nonhost plant, *Nicotiana benthamiana*, than the wild-type and constitutive-expression strains (Fig. 2C).

10.1128/mBio.02146-17.3 FIG S3  Verification of protein expression by immunoblotting. (A) Lon was constitutively overexpressed (OE) under the control of the P_*trp*_ promoter in the Δ*lon* mutant. Protein expression was detected with an anti-Flag antibody. (B) Expression of GFP and GFP chimeric proteins was confirmed by immunoblotting. An anti-RNA polymerase (RNAP) antibody was used to normalize the amount of gel loading. (C) LonS654D, LonS654E, and LonS654A were constitutively expressed under the control of the P_*trp*_ promoter. Protein expression was detected with an anti-Flag antibody. Download FIG S3, JPG file, 0.8 MB. Copyright © 2018 Zhou et al. 2018 Zhou et al. This content is distributed under the terms of the Creative Commons Attribution 4.0 International license.

The changes in pathogenicity in the host plants or the HR in the nonhost plant attributed to the deletion or overexpression of Lon may be ascribed to oscillated T3SS gene expression. In this regard, the expression of *hrpG*, *hrpX*, *hrpB1*, *hrcQ*, and *hrcU* was monitored by quantitative reverse transcription-PCR (qRT-PCR). HrpG and HrpX are two master regulators of T3SS in *X. citri* subsp. *citri* and are essential for bacterial virulence. HrcQ, HrcU, and HrpB1 are three *hrp*/*hrc* operons that are major T3SS components. We found that the expression levels of all of the genes tested were dramatically higher in the *lon* mutant than those in the wild type in both rich medium and the host (Fig. 2D). To further verify whether Lon regulates T3SS genes by altering *hrp*/*hrc* gene transcription, the promoters of *hrpB1*, *hrcU*, and *hrcQ* were fused with a β-glucuronidase (GUS) reporter gene. The resulting constructs were introduced into the wild-type and *lon* mutant strains. Histochemical staining of *X. citri* subsp. *citri* cells with 5-bromo-4-chloro-3-indolyl-β-d-glucuronide (X-Gluc) showed that all of the promoters tested had higher activity in the *lon* mutant than in the wild type (Fig. 2E). Similar results were obtained by quantification of GUS activity with *p*-nitrophenyl-β-d-glucuronide as a substrate (Fig. 2E). Taken together, our results indicate that Lon negatively regulates bacterial virulence by repressing T3SS gene transcription.

### Lon degrades HrpG in rich medium.

**FIG 3:**
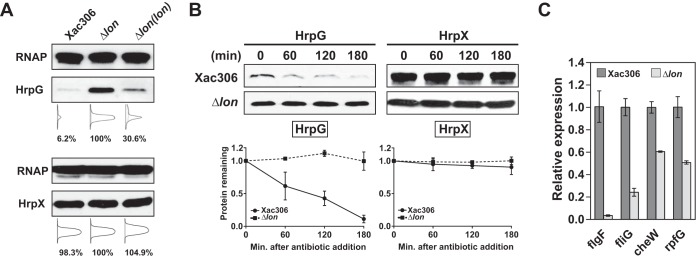
Lon degrades HrpG in rich medium. (A) HrpG and HrpX expression levels in wild-type strain 306, the Δ*lon* mutant, and the Δ*lon* mutant with chromosomal complementation were detected by immunoblotting. Densitometry plots were generated on the basis of the pixels of each band with ImageJ. The relative intensity of protein bands was calculated in percentages. RNAP, RNA polymerase. (B) *In vivo* degradation assays showing the stability of HrpG and HrpX. Epitope-tagged HrpG and HrpX were constitutively expressed under the control of P_*trp*_ in the wild type and the Δ*lon* mutant. Protein levels were monitored by immunoblotting (top). Band intensities were quantified (bottom), and error bars represent the standard deviations (*n* = 3). Statistical data of HrpG-6His are also shown in Fig. 4A. (C) Relative expression of genes specifically regulated by HrpG. Cells were incubated in rich medium overnight, and RNA samples were isolated. The expression of *flgF*, *fliG*, *cheW*, and *rpfG* was monitored by qRT-PCR. The mean values ± the standard deviations (*n* = 3) are plotted.

Control of *hrp*/*hrc* transcription in *Xanthomonas* spp. is mediated by the T3SS master regulators HrpG and HrpX. We therefore hypothesized that the global effect of Lon on *hrp*/*hrc* gene expression is due to proteolysis of these regulators. To test this hypothesis, plasmids constitutively expressing HrpG-6His and HrpX-Flag were transferred into the *lon* mutant and the wild type. To generate a Lon-complemented strain that is able to express HrpG-6His or HrpX-Flag, a sequence containing the full-length *lon* open reading frame (ORF) and its upstream native promoter was amplified and inserted into the chromosome of the *lon* mutant at the *amy* locus. The expression of HrpG-6His and HrpX-Flag was determined by immunoblotting. Interestingly, the HrpG-6His expression level in the *lon* mutant (100%) was significantly higher than that in the wild type (6.26%) or the *lon*-complemented strain (30.6%) (Fig. 3A). However, this expression pattern was not observed with HrpX-Flag.

To further investigate whether HrpG and/or HrpX are targeted by Lon, we determined the rate of HrpG-6His and HrpX-Flag degradation by using an *in vivo* degradation assay. To monitor *in vivo* degradation, translation was arrested in bacterial cultures by spectinomycin and the HrpX and HrpG protein levels were monitored and quantified at 60-min intervals. We found that HrpG-6His was not stable in the wild type but was stable in the *lon* mutant after protein synthesis shutoff. In the presence of Lon, HrpG-6His had a half-life of ~100 min (Fig. 3B). In contrast, HrpX-Flag was found to be stable in both the wild type and the *lon* mutant (Fig. 3B). These results support our notion that Lon targets HrpG but not HrpX.

Additionally, we monitored the expression levels of multiple genes (i.e., *figF*, *fliG*, *cheW*, and *rpfG*) that were previously shown to be negatively regulated by HrpG (8). The expression of all of the genes selected was downregulated in the *lon* mutant compared to that in the wild type (Fig. 3C), suggesting that deletion of Lon gives rise to a stabilized HrpG protein level. Taken together, our results suggest that, in rich medium, HrpG is an unstable protein whose degradation depends on the protease Lon.

### The N terminus of HrpG is recognized by Lon.

**FIG 4:**
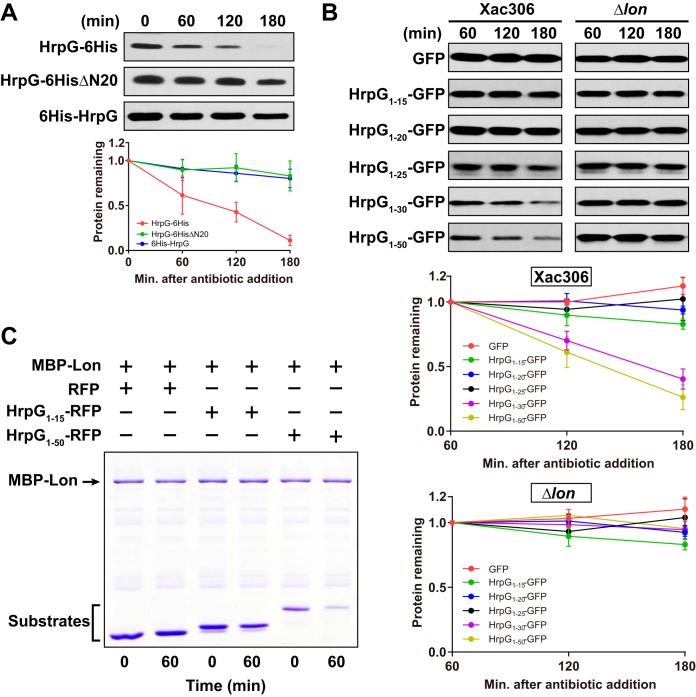
The N-terminal moiety of HrpG is required for Lon recognition. (A) Effect of the HrpG N-terminal moiety on its degradation capability. HrpG-6His, HrpG6HisΔN20, and 6His-HrpG were expressed in wild-type strain 306 under the control of P_*trp*_. Protein synthesis was blocked by the addition of 1 mg/ml spectinomycin. Samples collected at the time points indicated were analyzed by immunoblotting (top). Band intensities were quantified (bottom), and error bars represent the standard deviations (*n* = 3). Statistical data of HrpG-6His are also shown in Fig. 3B. (B) Determination of HrpG N-terminal amino acids that are responsible for Lon recognition. GFP and GFP chimeric proteins encoded in the pBRK plasmid were constitutively expressed in the wild type and the Δ*lon* mutant under the control of the P_*lac*_ promoter. Samples were collected at the time points indicated and subjected to immunoblotting (top). Band intensities were quantified (bottom), and error bars represent the standard deviations (*n* = 3). (C) *In vitro* protein degradation assay. Purified MBP-Lon_6_ (0.1 μM) was incubated with purified RFP (5 μM), HrpG_1-15_–RFP (5 μM), or HrpG_1-50_–RFP (3 μM) in the presence of 10 mM ATP at 28°C for 60 min. Samples were analyzed by SDS-PAGE, followed by Coomassie staining.

Lon degrades substrates through the recognition of a certain peptide sequence (also called a degradation tag or degron). The degradation tag is usually located at the N or C terminus of the substrate (31–33). We reasoned that the N terminus of HrpG may be responsible for Lon recognition because the attachment of a six-histidine tag to the C terminus of HrpG, before the HrpG stop codon, does not stabilize HrpG (Fig. 3B and 4A). To verify whether the N terminus of HrpG is recognized by Lon, we constructed 6His-HrpG by attaching a six-histidine tag after the HrpG start codon and HrpG-6HisΔN20 lacking the N-terminal 20 residues of HrpG. Both HrpG-6HisΔN20 and 6His-HrpG were significantly more stable than HrpG-6His (Fig. 4A). The data indicate that N-terminal deletion or fusion with a 6×His tag likely stabilizes HrpG by interfering with its recognition by Lon.

To identify HrpG N-terminal amino acids that are sufficient for Lon recognition, we constructed several green fluorescent protein (GFP) reporters with the HrpG N-terminal 15 to 50 amino acids. An empty vector expressing free GFP was used as a control. The expression of all constructs in the wild type and the *lon* mutant was verified by immunoblotting (Fig. S3B). We then performed the *in vivo* stability assay in NB medium for cells expressing different GFP reporters. In the absence of Lon, all chimeric GFP reporter proteins were stable. In the presence of Lon, however, HrpG_1-30_–GFP and HrpG_1-50_–GFP had a significantly greater degradation rate than other chimeric proteins (Fig. 4B). The half-lives of HrpG_1-30_–GFP and HrpG_1-50_–GFP were ~150 and ~130 min, respectively. We also observed that both the HrpG_1-30_–GFP and HrpG_1-50_–GFP proteins were less abundant in wild-type cells than in Δ*lon* mutant cells (Fig. S3A). These results suggest that the N-terminal 30 residues of the HrpG protein function as a degradation tag for *X. citri* subsp. *citri* Lon.

To examine whether the N terminus of HrpG is directly targeted by Lon substrate, degradation was further tested in an *in vitro* protein degradation assay. Lon fused to MBP (maltose binding protein) was purified from *E. coli* and incubated alongside HrpG_1-15_ fused to red fluorescent protein (RFP), HrpG_1-50_ fused to RFP, or RFP alone, and protein levels were visualized by Coomassie staining. RFP alone and the HrpG_1-15_–RFP fusion were stable in the presence of MBP-Lon, and protein levels were not significantly altered after a 60-min incubation. However, HrpG_1-50_–RFP protein levels were significantly reduced in the presence of MBP-Lon, indicating that this variant is directly degraded by Lon. Taking our results together, we conclude that the HrpG N terminus is responsible for recognition by the Lon protease.

### Phosphorylation of Lon is required for bacterial virulence and HR induction.

**FIG 5:**
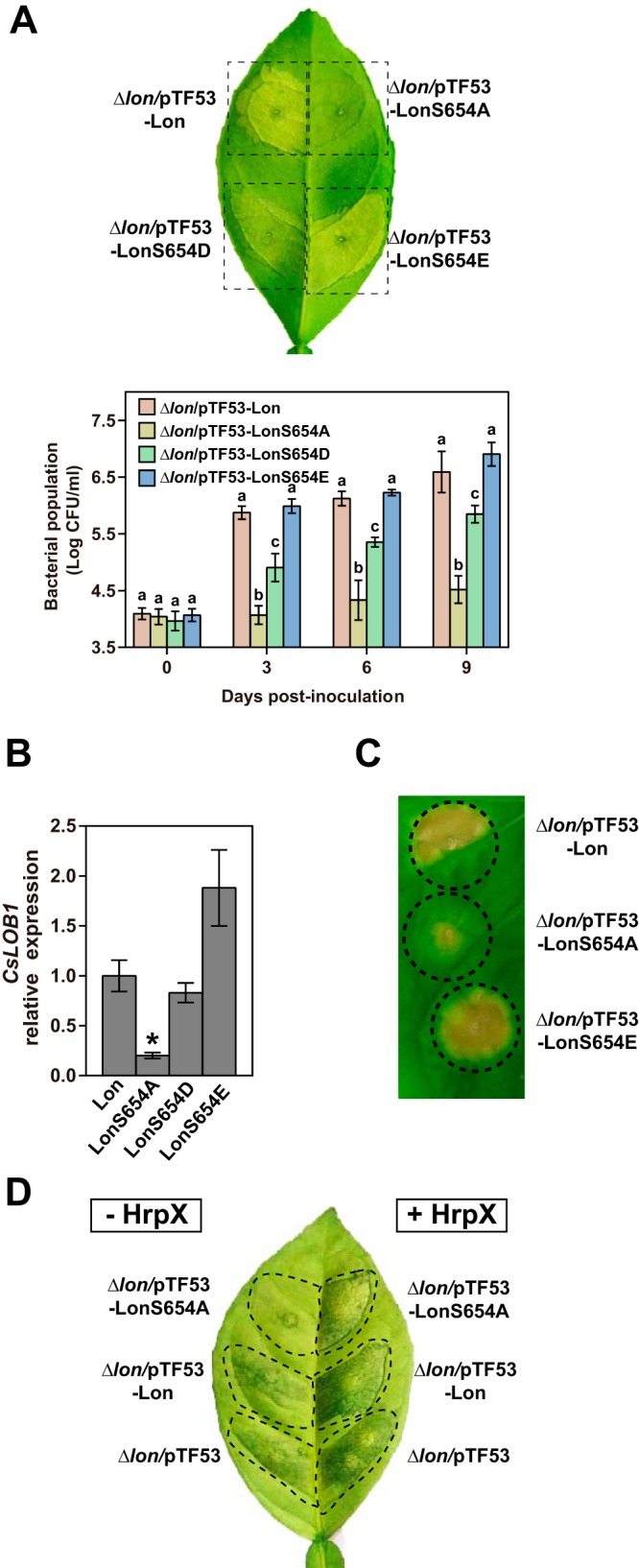
Phosphorylation of Lon protease is required for bacterial virulence and HR induction. (A) Δ*lon* mutant derivative strains expressing Lon, LonS654A, LonS654D, and LonS654E were inoculated onto sweet orange leaves at 10^6^ CFU ml^−1^. The inoculated leaves were photographed at 7 days postinoculation. The experiments were repeated three times with comparable results, and only one representative leaf is shown. Bacterial populations were quantified, and error bars represent the standard deviations (*n* = 3). The different letters indicate statistically significant differences determined by one-way analysis of variance (*P* < 0.01). (B) *CsLOB1* expression levels in grapefruit leaves inoculated with strains from panel A. *CsLOB1* expression was monitored by qRT-PCR at 48 h postinoculation. The mean values ± the standard deviations (*n* = 3) are plotted. The asterisk indicates a statistically significant difference (*P* < 0.01, Student *t* test). (C) Fully expanded 4-week-old *N. benthamiana* leaves were inoculated with Δ*lon* mutant derivative strains expressing Lon, LonS654A, and LonS654E at a concentration of 10^8^ CFU ml^−1^. HRs were observed and photographed at 1 week postinoculation. The experiments were repeated three times with comparable results, and only one representative leaf is shown. (D) Plasmids encoding constitutive expression of HrpX were introduced into Δ*lon* mutant derivative strains as indicated. Pustule formation by the resulting strains in the absence and presence of HrpX was compared. The inoculated leaves were photographed at 7 days postinoculation. The experiments were repeated three times with comparable results, and only one representative leaf is shown.

Given the fact that Lon is specifically phosphorylated in the host, we wondered whether host-induced Lon phosphorylation influences *X. citri* subsp. *citri* pathogenicity. To investigate the role of Lon phosphorylation in the regulation of bacterial virulence, we mutated the serine at Lon position 654 to aspartic acid (S654D) or glutamic acid (S654E). LonS654A is no longer able to be phosphorylated and is considered an unphosphorylated form (Fig. 1B); aspartic acid and glutamic acid act as phosphomimetic mutations and produce fully phosphorylated forms. Protein expression was confirmed by immunoblotting to rule out the influence of point mutations on protein stability (Fig. S3C). The *lon* mutant strains expressing wild-type Lon, LonS654A, LonS654E, and LonS654D were used to infiltrate sweet orange leaves with a needleless syringe at a bacterial titer of 10^6^ CFU ml^−1^. Interestingly, LonS654A failed to induce a canker pustule, unlike wild-type Lon, at 7 days postinoculation (Fig. 5A). However, ectopic expression of LonS654D or LonS654E in the *lon* mutant caused canker symptoms similar to those caused by a strain producing wild-type Lon. Consistent with the assay of pathogenicity in the host, the bacterial population of the *lon* mutant expressing LonS654A was significantly smaller than the population of cells expressing wild-type Lon, LonS654D, or LonS654E (Fig. 5A). It is noteworthy that LonS654D had partial complementation of the bacterial population, whereas full complementation was observed for LonS654E. This suggests that glutamic acid may better mimic phosphoserine than aspartic acid in this case.

To assess the effects of these Lon variants on T3E delivery, we also measured the expression levels of *CsLOB1* under the treatment of the *lon* mutant expressing different Lon varieties used in the pathogenicity assay. At 48 h postinoculation, *CsLOB1* expression induced by LonS654A was significantly lower than that induced by wild-type Lon, LonS654D, and LonS654E (Fig. 5B). Similarly, cells expressing LonS654E were able to induce a higher level of *CsLOB1* expression than cells expressing wild-type Lon and LonS654D, which agreed with the results of the pathogenicity assay and the bacterial population in the host. In addition, LonS654A failed to induce an HR in the nonhost plant *N. benthamiana*, while wild-type Lon and LonS654E triggered a typical HR at 7 days postinoculation (Fig. 5C). Our results show that phosphorylation of Lon at serine 654 is required for bacterial virulence and HR induction.

To address the question of whether Lon phosphorylation regulates bacterial virulence in an *hrp*/*hrc*-dependent manner, we overexpressed the *hrp*/*hrc* genes by constitutively expressing HrpX, which naturally acts downstream of HrpG, under the control of a *lac* promoter. Interestingly, we found that constitutive expression of HrpX could bypass the virulence defect caused by the LonS654A mutation and induce a canker pustule (Fig. 5D). Our data indicate that Lon phosphorylation is likely required to maintain *hrp*/*hrc* gene expression in the host.

### Phosphorylation of serine 654 in Lon protease inhibits HrpG degradation.

**FIG 6:**
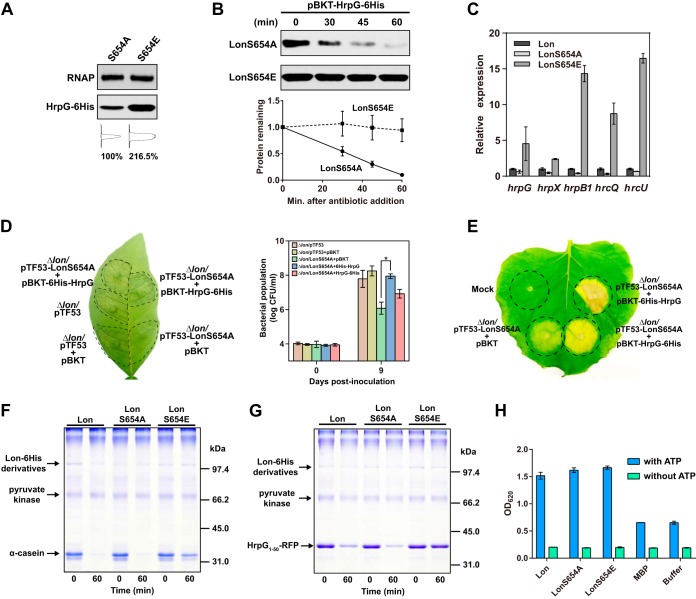
Lon protease phosphorylation at serine 654 inhibits HrpG degradation. (A) HrpG expression levels in the presence of LonS654A and LonS654E. Gel loading was normalized to the RNA polymerase (RNAP) β subunit. Densitometry plots were generated on the basis of the pixels of each band with ImageJ. The relative intensity of protein bands was calculated in percentages. (B) *In vivo* degradation assay showing HrpG stability in the presence of LonS654A and LonS654E. A plasmid encoding HrpG-6His was introduced into Δ*lon* mutant derivative strains expressing either LonS654A or LonS654E. The resulting strains were cultured overnight. Protein synthesis was blocked by the addition of spectinomycin at 1 mg/ml. Samples collected at the time points indicated were analyzed by immunoblotting (top). Band intensities were quantified (bottom), and error bars represent the standard deviations (*n* = 3). (C) Lon phosphorylation at serine 654 upregulates *hrp*/*hrc* gene expression. Strains expressing Lon, LonS654A, and LonS654E were cultured in rich medium overnight, and RNA samples were isolated. Relative *hrpG*, *hrpX*, *hrcQ*, *hrcU*, and *hrpB1* expression was monitored by qRT-PCR. Error bars represent the standard deviations (*n* = 3). (D) Stabilization of HrpG reverses the virulence defect caused by mutation at serine 654. Plasmid pBKT-HrpG-6His or pBKT-6His-HrpG was introduced into the Δ*lon*/LonS654A mutant. Δ*lon* mutant strains carrying empty vector pTF53 and/or pBKT served as positive controls. The Δ*lon/*LonS654A mutant strain carrying empty vector pBKT served as a negative control. Inoculated leaves were photographed at 7 days postinoculation. The experiments were repeated three times with comparable results, and only one representative leaf is shown. Bacterial populations were quantified, and error bars represent the standard deviations (*n* = 3). The asterisk indicates a statistically significant difference (*P* < 0.01, Student *t* test). (E) Evaluation of HR induction in the Δ*lon*/LonS654A mutant strain carrying the empty pBKT plasmid, pBKT-HrpG-6His, or pBKT-6His-HrpG. Strains were inoculated onto *N. benthamiana* leaves at 10^8^ CFU ml^−1^ resuspended in 10 mM MgCl_2_. HR induction was observed and photographed at 10 days postinoculation. The experiment was repeated three times with comparable results, and only one representative leaf is shown. Mock, 10 mM MgCl_2_. (F and G) *In vitro* protein degradation assay. Purified Lon, LonS654A, and LonS654E were incubated with α-casein (F) or HrpG_1-50_–RFP (G) at 28°C for 60 min in the presence of an ATP regeneration system. Samples were analyzed by SDS-PAGE, followed by Coomassie staining. (H) ATPase activities of purified Lon, LonS654A, and LonS654E in the presence or absence of 4 mM ATP. Purified MBP and reaction buffer were used as negative controls. The experiments were conducted in a 96-well microplate, and optical density (OD) at 620 nm was read. Error bars represent the standard deviations (*n* = 3).

The critical involvement of phosphorylation at serine 654 of Lon in *X. citri* subsp. *citri* virulence and HR induction suggests that phosphorylation of Lon may alter enzyme activity and inhibit HrpG degradation, thereby controlling T3SS expression in the host. To test this hypothesis, LonS654A and LonS654E were constitutively expressed in the *lon* mutant under the control of P_*trp*_. The HrpG protein level was higher in Δ*lon*/LonS654E than that in the Δ*lon*/LonS654A mutant (Fig. 6A). Additionally, we found that HrpG was significantly more stable when phosphomimetic Lon was present. The half-life of HrpG dropped to ~35 min in the Δ*lon*/LonS654A mutant (Fig. 6B). Additionally, the expression of five representative *hrp*/*hrc* genes, i.e., *hrpG*, *hrpX*, *hrpB1*, *hrcQ*, and *hrcU*, was significantly induced in Δ*lon*/LonS654E mutant cells grown in rich medium (T3SS repressing) (Fig. 6C). Such induction was not observed in Δ*lon*/LonS654A or Δ*lon* mutant cells. These results support the conclusion that Lon protease phosphorylation attenuates HrpG degradation *in vivo*.

We wondered whether artificially increasing HrpG protein abundance in the Δ*lon*/LonS654A mutant could restore pathogenicity. To address this question, a plasmid encoding either 6His-HrpG or HrpG-6His was introduced into the Δ*lon*/LonS654A mutant. As revealed by a bacterial population assay in the host, the 6His-HrpG construct fully complemented the virulence defect to the Δ*lon* level but the HrpG-6His construct did not (Fig. 6D). However, we observed a slight increase in pathogenicity and the population of bacterial cells harboring HrpG-6His. This is probably due to overproduced HrpG-6His protein and its incomplete proteolysis. In addition, we tested how expressing 6His-HrpG and HrpG-6His in the presence of LonS654A might affect HR development in *N. benthamiana*. The Δ*lon*/LonS654E mutant strain carrying the 6His-HrpG construct induced a stronger HR than that that carrying HrpG-6His (Fig. 6E), which is consistent with our previous finding that the degradation tag is located at the HrpG N terminus.

To further solidify our findings, we attempted to reconstitute Lon-mediated HrpG degradation *in vitro*. In a protein degradation assay, the known Lon substrate α-casein and HrpG_1-50_–RFP (Fig. 4D), which was identified in this study, were tested. Both α-casein and HrpG_1-50_–RFP were degraded by wild-type Lon and LonS654A. However, the degradation of α-casein and HrpG_1-50_–RFP was impaired in the presence of LonS654E (Fig. 6F and G). We ruled out the possibility that impaired proteolytic activity of LonS654E is due to substitution-induced inactivation of the ATPase domain. The ATPase activity assay showed that mutation of serine to alanine or glutamic acid did not alter ATP hydrolytic activity (Fig. 6H). Taken together, our results indicate that phosphorylation of serine 654 of the Lon protease stabilizes the HrpG protein, thereby conferring bacterial virulence in the host.

## DISCUSSION

Our genetic and biochemical experiments provide evidence that the Lon protease regulates T3SS gene expression and bacterial virulence through distinct proteolysis of the master regulator HrpG. We show that, in rich medium, Lon represses T3SS by robust degradation of HrpG by recognizing its N-terminal 50 amino acids, thus repressing T3SS expression. Host-induced phosphorylation, however, deactivates Lon proteolytic activity, resulting in increased stability of HrpG and bacterial virulence. Specifically, ectopic expression of LonS654A (dephosphorylated form) in the Δ*lon* mutant fails to induce canker symptoms and expression of the canker susceptibility gene *CsLOB1* in citrus, as well as a nonhost HR, whereas LonS654D and S654E (phosphomimetic mutations) cause canker symptoms and induce *CsLOB1* and an HR. Similarly, LonS654A robustly degrades HrpG and suppresses T3SS gene expression, whereas LonS654E stabilizes HrpG and enhances T3SS gene expression. Constitutive expression of either an undegradable form of HrpG or HrpX, which is tightly regulated by HrpG (8), in the Δ*lon* mutant containing LonS654A restores virulence, further supporting the idea that Lon regulates T3SS gene expression and virulence via degradation of HrpG. Therefore, Lon is most likely a central switch for T3SS gene expression in the host and in non-T3SS-inducing environments.

Proteolysis mediated by the Lon protease is an energy-dependent process. The inhibition of Lon degradation capacity is ascribed to phosphorylation at serine 654 rather than compromised ATPase activity. Phosphorylation of serine 654 may induce a subtle conformational change in the Lon P domain, resulting in a closed or narrowed degradation chamber during the transit of the substrate through the proteolytic domain. Since Lon forms either hexamers or dodecamers (21, 22, 34), we speculate that all Lon subunits need to be dephosphorylated to keep full proteolytic activity, whereas the phosphorylation of one or more subunits may sufficiently interfere in proteolytic activity. It remains to be determined how dephosphorylated Lon recognizes HrpG for degradation despite knowing that the N-terminal 30 amino acids are critical for this recognition. We noted that the phosphorylation/dephosphorylation status of Lon affects its proteolytic activity not only on HrpG but also on α-casein, a known Lon target. Thus, phosphorylation of Lon may differentially control many molecular processes of bacteria in many different environments. It was reported that a redox switch regulates *E. coli* Lon protease activity between aerobic and anaerobic conditions in a similar way by altering the diameter of the degradation channel (35). Cysteines 617 and 691 usually form a disulfide bond on the Lon protease domain surface. In anaerobic environments, the disulfide bond is reduced, which leads to ~30% narrowing of the exit pore and an ~80% decrease in enzyme activity; in turn, the oxidized disulfide bond in aerobic environments restores the narrowed pore size and enzyme activity. Therefore, specific Lon protease phosphorylation in the host environment likely acts as a switch to turn on and off the expression of infection machinery, such as T3SS, and physiological processes according to various stimuli.

Phosphorylation has been known to affect the activity of other proteases. HtrA2/Omi is a serine protease released from mitochondria upon apoptosis that has been implicated in Parkinson’s disease (36). Detailed studies revealed that serine/threonine kinases Akt1 and Akt2 are able to phosphorylate HtrA2/Omi at serine 212 within its proteolytic domain both *in vivo* and *in vitro* (37). Phosphorylation of HtrA2/Omi attenuates the protease activity and inhibits the release of HtrA2/Omi from mitochondria. In addition, large-scale phosphoproteomic studies identified a ubiquitin-specific protease, USP1, phosphorylated at serine 313 (38). The catalytic activity of USP1 requires a specific interaction with a WD40 repeat protein, UAF1. Further experiments showed that USP1 phosphorylation is essential for interaction with UAF1 and its protease activity (38). Therefore, it is conceivable that serine phosphorylation might be a common mechanism in the regulation of protease activity. Interestingly, Lon protease phosphorylation was also found in the human pathogen *Acinetobacter baumannii*, implying that phosphorylation on Lon protease might be a conserved mechanism in the regulation of enzymatic activity in both plant and human pathogens (39). Differences in the distribution of bacterial protein phosphorylation in host versus nonhost environments appear to be a common phenomenon in stress adaptation (40).

**FIG 7:**
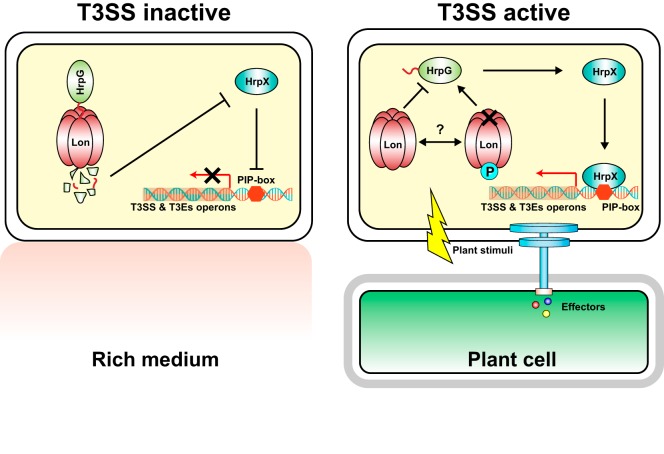
Proposed model of a phosphorylation switch on Lon protease that regulates bacterial T3SS in host and nonhost environments. PIP, plant-inducible promoter.

These observations lead us to propose a model in which the bacterial T3SS is differently controlled by Lon protease (Fig. 7). When *X. citri* subsp. *citri* cells are grown in non-T3SS-inducing environments, e.g., rich medium, Lon stays predominantly in a dephosphorylated form and maintains its proteolytic activity. The HrpG protein is targeted by Lon through an N-terminal degron and is degraded. The degradation of HrpG results in the downregulation of HrpX and the *hrp*/*hrc* genes. Therefore, the T3SS is turned off in rich medium. Conversely, when *X. citri* subsp. *citri* cells infect the host, plant stimuli can be perceived and activate corresponding serine/threonine kinases, which catalyze Lon protease phosphorylation at serine 654. Consequently, an increased proportion of phosphorylated Lon attenuates Lon proteolytic activity and protects HrpG from degradation. Enhanced stability of HrpG upregulates HrpX and turns on the bacterial T3SS. The intricate environment of the host may contribute to the dynamic balance between phosphorylation and dephosphorylation of Lon, affecting the stability of HrpG and downstream transcription of T3SS. Inhibition of Lon phosphorylation at serine 654 might be a plausible way to control bacterial diseases. In addition, LonS654A has shown superior proteolytic activity, e.g., against HrpG. LonS654A has potential for application against bacterial pathogens of plants and animals. The discovery of the control mechanism of T3SS suppression in non-T3SS-inducing environments and induction of T3SS via Lon phosphorylation in the host represent a conceptual advance in addressing a fundamental question of T3SS expression. HrpG and Lon are conserved in all *Xanthomonas* spp. However, compared to the wide distribution of Lon, HrpG is limited to several bacterial genera, including *Xanthomonas*, *Burkholderia*, and *Ralstonia* (6, 41, 42). Lon controls T3SS genes via different T3SS regulators in other bacteria that do not contain HrpG homologues. In fact, Lon is known to control T3SS regulation in *Salmonella* by targeting HilC and HilD, which regulate the SPI1 T3SS transcriptional activator HilA (43). In *P. syringae*, Lon regulates T3SS expression by targeting the HrpR protein, which regulates HrpL, an alternative sigma factor controlling T3SS (24). It remains to be determined whether phosphorylation of Lon controls the induction of T3SS, via degradation of critical T3SS regulators, in other bacteria in the host compared to non-T3SS-inducing environments.

## MATERIALS AND METHODS

### Bacterial strains, plasmids, and primers.

The bacterial strains and plasmids used in this study are listed in Table S3. The primers used in this study are listed in Table S4. *Xanthomonas* strains were routinely grown at 28°C in rich (NB) medium or nutrient agar (NA) plates. *E. coli* strains were grown at 37°C in Luria-Bertani (LB) liquid medium or on LB agar plates. Antibiotics were used at the following concentrations for both *E. coli* and *Xanthomonas*: rifamycin, 50 μg/ml^−1^; kanamycin, 50 μg/ml^−1^; ampicillin, 100 μg/ml^−1^; gentamicin, 5 μg/ml^−1^.

10.1128/mBio.02146-17.6 TABLE S3  Strains and plasmids used in this study. Download TABLE S3, DOCX file, 0.02 MB. Copyright © 2018 Zhou et al. 2018 Zhou et al. This content is distributed under the terms of the Creative Commons Attribution 4.0 International license.

10.1128/mBio.02146-17.7 TABLE S4  Primers used in this study. Download TABLE S4, DOCX file, 0.01 MB. Copyright © 2018 Zhou et al. 2018 Zhou et al. This content is distributed under the terms of the Creative Commons Attribution 4.0 International license.

To create *lon* mutant strains, two PCR products extending ~800 bp either upstream or downstream of the *lon* gene were amplified by using genomic DNA and cloned into a pNPTS138 suicide vector. The deletion vector was transformed into wild-type *Xanthomonas* sp. strain 306, and gene deletion was conducted by a two-step *sacB* counterselection procedure (44). To generate *lon*-complemented strains, the entire *lon* ORF and its 320-bp native promoter were amplified and cloned into a pPM7g vector, which contains an α-amylase gene allele allowing for single-crossover recombination on the chromosome. Transformant selection was performed as previously described (44).

### Analysis of the phosphorylation status of Lon by liquid chromatography-tandem mass spectrometry (LC-MS/MS).

*X. citri* subsp. *citri* cells were incubated in NB medium at 28°C to stationary phase. Inoculated citrus leaves were cut into thin strips and immerged in 50 ml of distilled H_2_O. Bacterial cells were extracted by rigorous vortexing for 5 min, and the supernatant was filtered with two layers of lens paper. Cells collected from NB medium and plant leaves were pelleted at 4°C and immediately frozen in liquid nitrogen. Harvested cells were resuspended in lysis buffer containing 0.1 M Tris-HCl (pH 8.0), PhosSTOP phosphatase inhibitor cocktail (Roche), and protease inhibitor cocktail (Sigma). Samples were sonicated for 5 min with 3-s on and off pulses. Supernatants were collected by centrifugation at 12,000 rpm for 10 min at 4°C, reduced with dithiothreitol (DTT), and alkylated with iodoacetamide. Protein samples were further purified with a PD-10 desalting column (GE Healthcare) and digested with trypsin overnight at 37°C. Phosphopeptide enrichment was done with TiO_2_ Toptip microcolumns (GlygenSci, Columbia, MD) as described previously (45).

The lyophilized peptides were resuspended in 20 μl of loading buffer (3% acetonitrile, 0.1% acetic acid, 0.01% trifluoroacetic acid) and loaded onto a C_18_ capillary trap cartridge (LC Packings). Samples were then separated on a 15-cm nanoflow analytical C_18_ column (PepMap, 75-mm inside diameter, 3 μm, 100 Å) at a flow rate of 300 nl/min on a nanoLC ultra 1D plus system (AB Sciex). Solvent A was 3% (vol/vol) acetonitrile (ACN) and 0.1% (vol/vol) acetic acid, and solvent B was 97% (vol/vol) ACN and 0.1% (vol/vol) acetic acid. Peptide separation was performed with a linear gradient of 3 to 40% solvent B for 20 min, followed by an increase to 90% solvent B in 5 min and held for 5 min. The flow was directly sprayed to an LTQ Orbitrap-XL mass spectrometer (Thermo Fisher, Bremen, Germany). MS^2^ spectra were acquired in a data-dependent mode. An Orbitrap full MS scan (resolution, 3 × 10^4^; mass range, 400 to 1,800 Da) was followed by 10 MS2 scans in the ion trap, which were performed via collision-induced dissociation on the 10 most abundant ions. The isolation window for ion selection was 3 Da. The normalized collision energy was set at 28%, and the dynamic exclusion time was 20 s (46). Additionally, if a phosphate neutral loss of 98, 49, 32.66, and 24.5 *m*/*z* below the precursor ion mass was detected, there was an additional activation. This multistage activation event was repeated for the top 10 ions in a data-dependent manner, provided the precursor exceeded a threshold of 500 ion counts (47).

All MS/MS samples were analyzed with Mascot version 2.4.1 (Matrix Science, Inc., London, United Kingdom). Mascot was set up to search the Xanthomonas_306 database (4,429 entries) assuming the digestion enzyme trypsin. Mascot was searched with a fragment ion mass tolerance of 0.80 Da and a parent ion tolerance of 10.0 ppm. Carbamidomethylation of cysteine was specified in Mascot as a fixed modification. Deamidation of asparagine and glutamine, oxidation of methionine, and phosphorylation of serine, threonine, and tyrosine were specified in Mascot as variable modifications. Scaffold (version Scaffold_4.3.4; Proteome Software Inc., Portland, OR) was used to validate MS/MS-based peptide and protein identifications. Peptide identifications were accepted if they could be established at >25.0% probability by the Peptide Prophet algorithm (48) with Scaffold delta-mass correction. Protein identifications were accepted if they could be established at >5% probability. In addition, the fragmentation spectra were manually checked for b and y ion series coverage (49).

### RNA extraction and qRT-PCR.

RNA extraction and qRT-PCR were performed as described previously (44). Gene expression was normalized to the *gyrA* gene in *X. citri* subsp. *citri* and the gene for glyceraldehyde-3-phosphate dehydrogenase in citrus as endogenous controls. Fold changes were calculated by the 2^−ΔΔ*CT*^ method (50).

### Pathogenicity assay, HR assay, and bacterial population.

*X. citri* subsp. *citri* cells with a titer of 10^6^ or 10^8^ CFU ml^−1^ were used to infiltrate grapefruit (*Citrus paradisi* Macfad. cv. Duncan) or sweet orange [*Citrus sinensis* (L.) Osbeck] leaves with needleless syringes. Inoculated plants were kept in a quarantined greenhouse and photographed 3 to 7 days postinoculation. HR assays were performed with *N. benthamiana* as described previously (44). For bacterial population assays in plants, three independent leaf discs were collected with a leaf puncher (1 cm in diameter) and ground in 1 ml of tap water. Serial dilutions of cell suspensions were plated on NA solid medium for CFU counting.

### Protein purification and modeling.

Protein purification was performed in accordance with the instructions supplied with the pMAL Protein Fusion and Purification System (NEB) and The QIA*expressionist* (Qiagen, Valencia, CA), with modifications. Briefly, the full-length ORF of *lon* was cloned into pMAL-c5x and pBbE1a-RFP to generate pMAL-Lon and pBbE1a-Lon, respectively. The Q5 Site-Directed Mutagenesis kit was used to generate S654A, S654D, and S654E mutations. Exhaustive attempts to produce MBP-LonS654A were unsuccessful. The recombinant proteins harboring Lon point mutations were purified by using a histidine tag.

The recombinant plasmids were introduced into BL21 Star (DE3)pLysS (Invitrogen). A 500-ml culture of *E. coli* cells was grown at 37°C in LB medium (with the addition of 0.2% glucose for MBP purification) to an optical density at 600 nm of 0.8 and then incubated with 1 mM isopropyl-β-d-thiogalactopyranoside (IPTG) for 2.5 h. Cells were sonicated in purification buffer (50 mM Tris-HCl [pH 8.0], 200 mM NaCl, 1 mM EDTA, 1 mM DTT). For purification of MBP-Lon, cell lysate was incubated with amylose resin for 20 min. The amylose column was washed four times with 10 volumes of purification buffer. Protein was eluted in purification buffer supplied with 10 mM maltose and 20% glycerol. Control MBP was purified by the same procedure. For purification of histidine recombinant protein, crude extract from *E. coli* cells was incubated with Ni-nitrilotriacetic acid agarose for 30 min. The agarose beads were then washed with 10 volumes of purification buffer with the addition of 20 mM imidazole. The protein was eluted in purification buffer containing 300 mM imidazole. LonS654A, LonS654E, RFP, HrpG_1-15_–RFP, and HrpG_1-50_–RFP were purified by the same procedure.

The structure of the proteolytic domain of *X. citri* subsp. *citri* Lon was built by using the published *E. coli* Lon structure (PDB code 3WU4) as a template. Images were produced with PyMOL software (http://www.pymol.org).

### Immunoprecipitation and Phos tag gel analysis.

For immunoprecipitation of Lon, 100-ml *X. citri* subsp. *citri* bacterial cultures containing Lon or LonS654A overexpression plasmids were grown overnight at 28°C in rich medium (pH 6.3), pelleted, and resuspended in Tris-buffered saline supplemented with MS-SAFE protease and phosphatase inhibitor mix (Sigma). Samples were lysed by sonication and centrifuged at 14,000 rpm for 10 min at 4°C. Supernatants were collected, incubated overnight with *E. coli* Lon protease polyclonal antibody (Biorbyt) at 4°C, and then incubated with EZview red protein G affinity gel (Sigma) for 1 h. Samples were subjected to phosphate affinity SDS-PAGE with acrylamide-pendant Phos tag (Wako) in accordance with the manufacturer’s instructions and immunoblotted with anti-Lon protease antibody.

### Protein *in vivo* and *in vitro* degradation assays.

For *in vivo* stability assay, 20 ml of an overnight *X. citri* subsp. *citri* culture was pelleted and resuspended in 10 ml of fresh medium for an additional 1-h incubation. After incubation, a spectinomycin solution was directly added to the culture at a final concentration of 1 mg/ml to block protein synthesis. A 1-ml aliquot was harvested at each time point and lysed in protein loading buffer; this was followed by boiling for 5 min. The samples were subjected to SDS-PAGE and immunoblotting. The SDS-PAGE gel was transferred onto a polyvinylidene difluoride membrane (Millipore) with a Trans-Blot Turbo system (Bio-Rad, Hercules, CA). The membrane was blocked with 5% dry milk in TBST buffer (50 mM Tris, 150 mM NaCl, 0.05% Tween 20 [pH 7.6]). The membrane was incubated with a primary antibody and a secondary antibody for 1 h at room temperature. Immunoactive proteins were detected with an ECL detection kit and X-ray film. Protein bands were quantified with ImageJ software. Statistical analyses were plotted with PRISM (GraphPad Inc., San Diego, CA).

For *in vitro* degradation assays, reaction mixtures contained 0.1 μM Lon_6_, 5 μM substrate, and 10 mM ATP or an ATP regeneration system (rabbit muscle pyruvate kinase [10 U ml^−1^; Sigma], 20 mM phosphoenolpyruvate [Sigma], 4 mM ATP) in Lon degradation buffer (100 mM NaCl, 10 mM MgCl_2_, 1 mM DTT, 25 mM Tris-HCl [pH 8.0]). Reactions were carried out at 37°C, and samples were taken at appropriate time points, as indicated in the figures. SDS loading dye was used to quench the reaction. Samples were separated by SDS-PAGE, and gels were stained with Coomassie blue G-250.

### GUS assay.

Histochemical staining of *X. citri* subsp. *citri* cells and quantitative GUS activity assays were performed as described previously (44).
