# Supplementary figures and images for: A Phosphorylation Switch on Lon Protease Regulates Bacterial Type III Secretion System in Host
Source: mBio. 2018 Jan 23;9(1):e02146-17. doi: 10.1128/mBio.02146-17 (PMC5784255; doi:10.1128/mBio.02146-17)

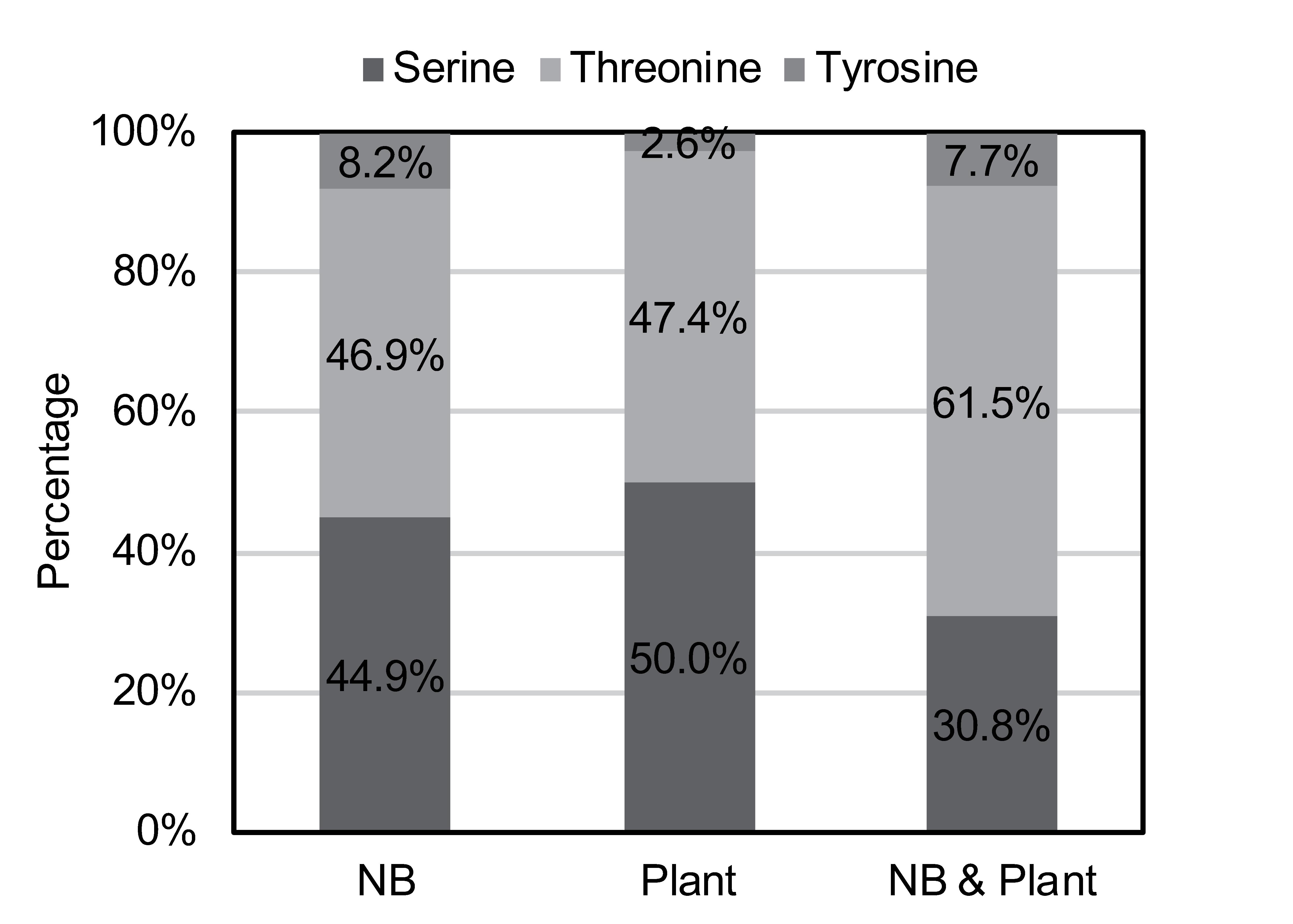

Supplement: FIG S1 [file mbo001183690sf1.jpg]

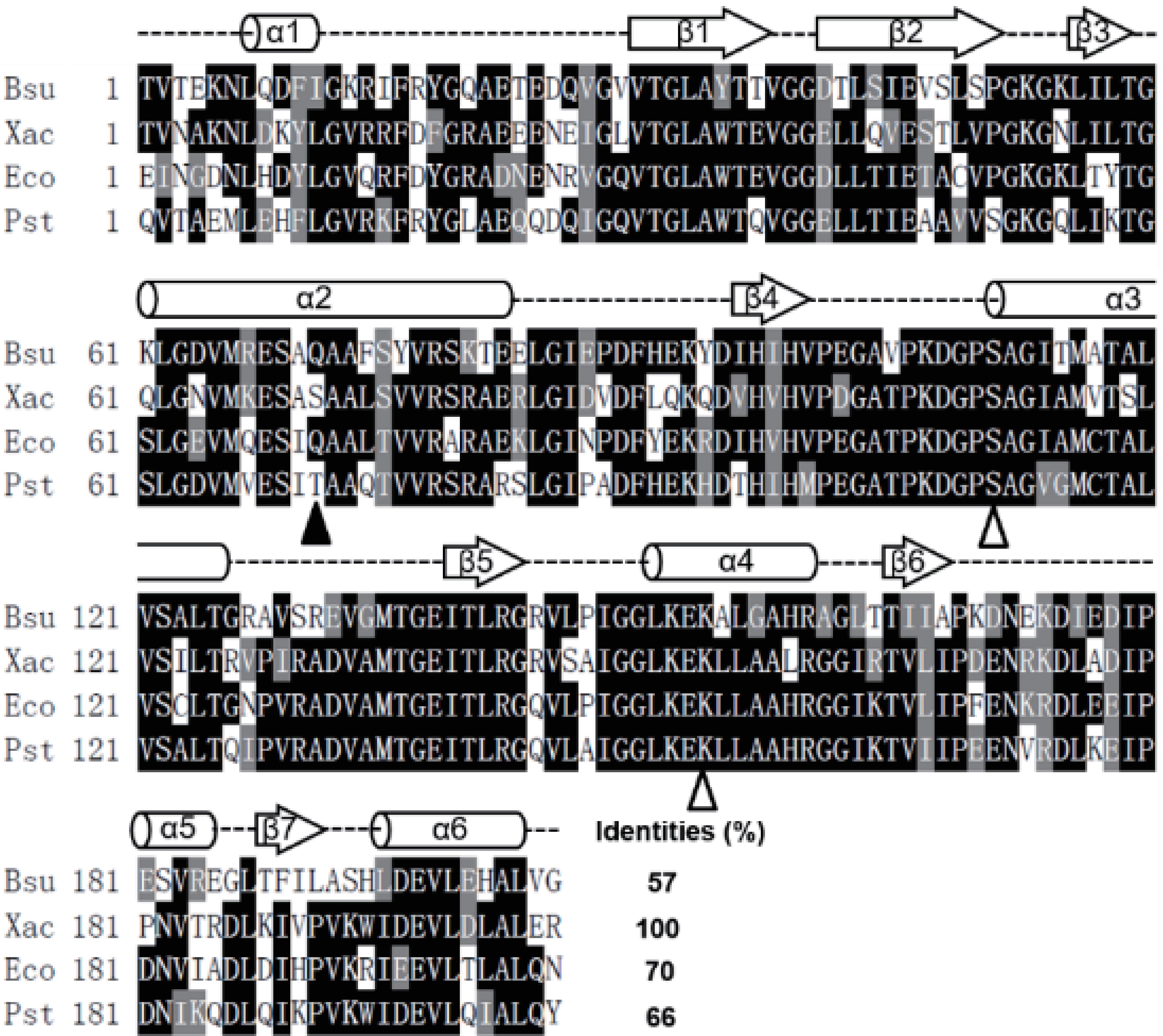

Supplement: FIG S2 [file mbo001183690sf2.jpg]

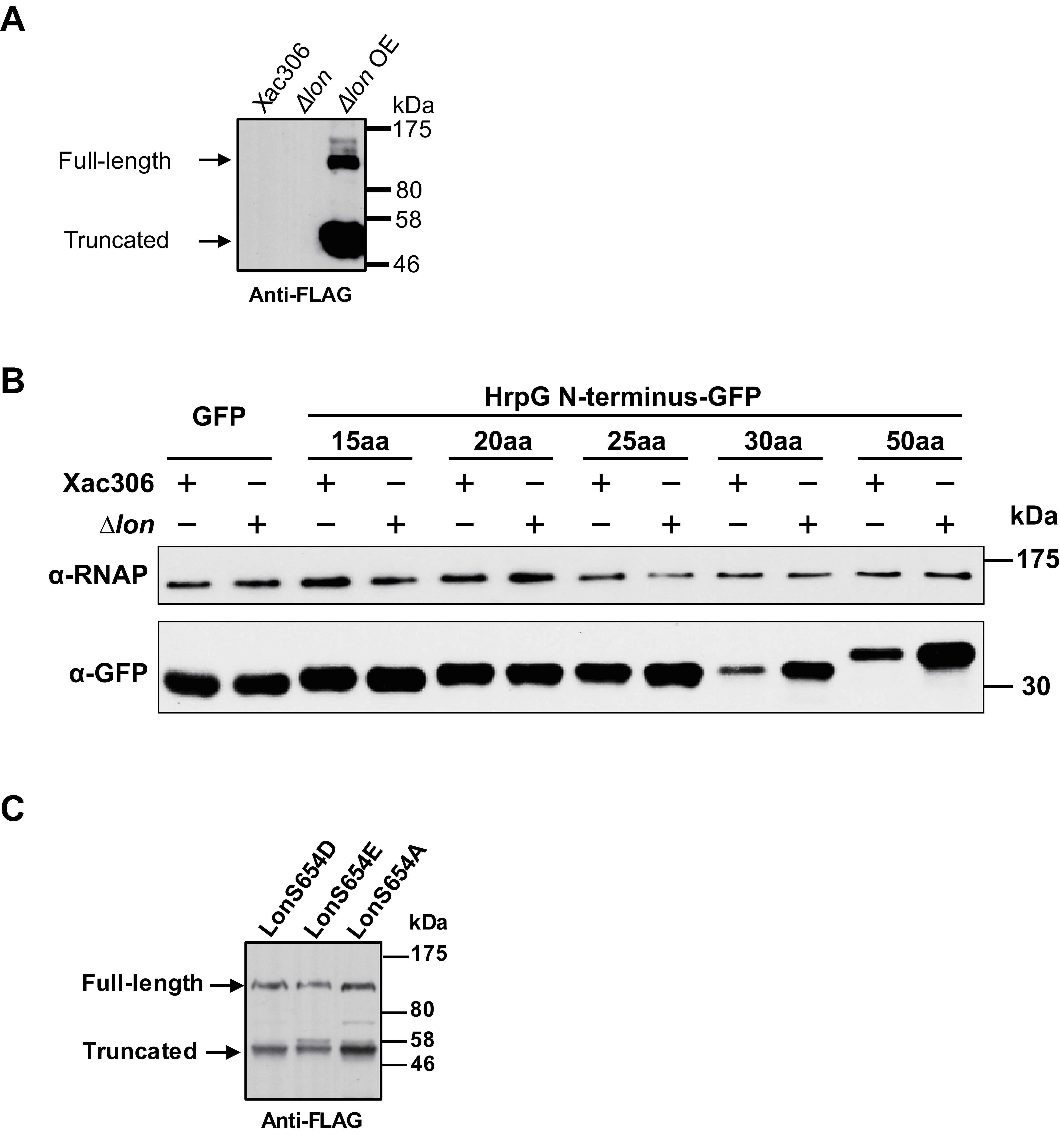

Supplement: FIG S3 [file mbo001183690sf3.jpg]
