# Supplementary material for: A Phosphorylation Switch on Lon Protease Regulates Bacterial Type III Secretion System in Host
Source: mBio. 2018 Jan 23;9(1):e02146-17. doi: 10.1128/mBio.02146-17 (PMC5784255; doi:10.1128/mBio.02146-17)
Supplement: TABLE S1 [file mbo001183690st1.docx]

| **Table S1**. List of identified phosphopeptides from *Xanthomonas citri* subsp. *citri* | | | | | | |
| --- | --- | --- | --- | --- | --- | --- |
| Acc. (GI) No | Gene locus | Gene name | Protein description | Phosphorylated peptide | NB^(a)^ | Plant^(b)^ |
| **Energy production and conversion** | | | | | | |
| 21106547 | XAC0460 | phaD | monovalent cation/H+ antiporter subunit D | ApTTPQRR | ✗ | ✔ |
| 21106979 | XAC0850 | ssuD | sulfonate monooxygenase | MAALHpSRGQR | ✗ | ✔ |
| 21107188 | XAC1046 | icd | isocitrate dehydrogenase | SPLTTPVGEGFSpSINVAMR | ✔ | ✔ |
|  |  |  |  | SPLTTPVGEGFpSSINVAMR | ✔ | ✔ |
| 21107867 | XAC1675 | dsbE | C-type cytochrome biogenesis protein/thioredoxin | HIGVLpTPDAINDELLPAIApSLPK(*) | ✔ | ✔ |
| 21108982 | XAC2701 | nuoD | NADH-ubiquinone oxidoreductase NQO4 subunit | NHLMWVGpSNALDLGAMAVMLYAFR | ✔ | ✗ |
| 21110033 | XAC3650 | atpG | ATP synthase gamma chain | MVAMKAASDNANKMIGTLQLVpYNK | ✔ | ✗ |
| 21110506 | XAC4089 | - | halogenase | MNAQRADVVIpTGGGLAGLpSLALQLKQR(*) | ✗ | ✔ |
|  |  |  |  |  |  |  |
| **Cell cycle control, cell division** | | | | | | |
| 21108487 | XAC2247 | - | conserved hypothetical protein | SRELNYHApTNAAAALALK | ✔ | ✗ |
|  |  |  |  |  |  |  |
| **Amino acid transport and metabolism** | | | | | | |
| 21109214 | XAC2911 | lysA | bifunctional diaminopimelate decarboxylase/asparta | VNTGGKASKFGLSpSpTR(*) | ✔ | ✗ |
|  |  |  |  |  |  |  |
| **Carbohydrate transport and metabolism** | | | | | | |
| 21107970 | XAC1772 | - | conserved hypothetical protein | SLMpSMHSPVARVIASSPAR | ✔ | ✗ |
| 21108267 | XAC2041 | ppsA | phosphoenolpyruvate synthase | GGRpTCHAAIIAR | ✔ | ✔ |
|  |  |  |  | pTCHAAIIAR | ✔ | ✔ |
| 21109955 | XAC3579 | xanA | phosphoglucomutase | AAGGVMVTApSHNPMDYNGMK | ✔ | ✔ |
|  |  |  |  | AAGGVMVpTASHNPMDYNGMK | ✔ | ✔ |
| 21110666 | XAC4231 | - | glucan 1,4-beta-glucosidase | LDRpTpTIATDGSLAATVTVK(*) | ✔ | ✗ |
|  |  |  |  |  |  |  |
| **Coenzyme transport and metabolism** | | | | | | |
| 21106726 | XAC0622 | hemC | porphobilinogen deaminase | pTpTLRIATR(*) | ✗ | ✔ |
| 21106928 | XAC0804 | sahH | adenosylhomocysteinase | GYELENGSpTWVDEPASSHEEGVIKALLK | ✔ | ✗ |
| 21106941 | XAC0815 | - | methyltransferase | LLETTLFNQIDYSGARRLLEVGSGVGAQpTEILLR | ✔ | ✗ |
| 21109025 | XAC2742 | btuB | TonB-dependent receptor | LRGpTLAQAVR | ✔ | ✗ |
| 21109806 | XAC3447 | thiC | thiamine biosynthesis protein | MNAAPpTVLQQQAQSLSEAVTQPIPGSRK | ✔ | ✗ |
|  |  |  |  |  |  |  |
| **Lipid transport and metabolism** | | | | | | |
| 21108237 | XAC2013 | fadB | 3-hydroxyacyl-CoA dehydrogenase | VSNpSAVNAK | ✗ | ✔ |
| 21108343 | XAC2118 | - | conserved hypothetical protein | TATAPpTVR | ✗ | ✔ |
| 21109371 | XAC3054 | fadE | acyl-CoA dehydrogenase | FKESLSGRLGDVLpSHIpYMTSAILK(*) | ✔ | ✗ |
| 21110709 | XAC4270 | plsB | glycerol-3-phosphate acyltransferase | RSSSNpSLIPEQRGGK | ✔ | ✗ |
|  |  |  |  |  |  |  |
| **Translation, ribosomal structure and biogenesis** | | | | | | |
| 21107095 | XAC0961 | rplK | 50S ribosomal protein L11 | STPApSVLLKK | ✔ | ✗ |
| 21107098 | XAC0964 | rplL | 50S ribosomal protein L7/L12 | EAKDLpTEAGGMLKEGASK | ✔ | ✗ |
| 21107109 | XAC0973 | rplD | 50S ribosomal protein L4 | ELVITGpSNNK | ✔ | ✗ |
| 21107110 | XAC0974 | rplW | 50S ribosomal protein L23 | VSEKpTAR | ✗ | ✔ |
| 21110768 | XAC4327 | uahA | urea amidolyase | TVQGFICEpSWATRGAEDITALGGWR | ✔ | ✗ |
|  |  |  |  |  |  |  |
| **Transcription** | | | | | | |
| 21106597 | XAC0508 | - | transcriptional regulator lysR family | QRQQYEVpTHADLVRELVAANLGVGIVPQTMAGAMR | ✔ | ✗ |
| 21107101 | XAC0966 | rpoC | RNA polymerase beta' subunit | CYGRDLARGHQVNIGEAVGVIAAQpSIGEPGTQLTMR | ✔ | ✗ |
| 21107615 | XAC1443 | yybA | transcriptional regulator marR family | MNVSSSApTPpSFGLLLR(*) | ✗ | ✔ |
|  |  |  |  |  |  |  |
| **DNA Replication, recombination and repair** | | | | | | |
| 21110002 | XAC3622 | dinP | DNA polymerase IV | TQIREEpTQLTASAGIAPNK | ✔ | ✗ |
| 21110092 | XAC3704 | - | DNA polymerase related protein | AVQRDpTHKMK | ✔ | ✗ |
|  |  |  |  |  |  |  |
| **Cell wall/membrane/envelope biogenesis** | | | | | | |
| 21106063 | XAC0023 | ctp | carboxyl-terminal protease | SNPGGLLTpSAVQVADDLLDKGNIVSTR | ✔ | ✗ |
| 21109739 | XAC3386 | mrcA | penicillin-binding protein 1A | FNRApTQAR | ✗ | ✔ |
|  |  |  |  |  |  |  |
| **Cell motility** | | | | | | |
| 21108194 | XAC1975 | fliC | flagellar protein | MAQVINpTNVMSLNAQR | ✔ | ✗ |
| 21109423 | XAC3099 | pilJ | pilus biogenesis protein | NPAALSALEQpSQAQWTTMKK | ✔ | ✗ |
| 21109883 | XAC3515 | bcsC | cellulose synthase subunit C | ALAGSpTAAAQQpTPTGLADTLNAMAASGNGSR(*) | ✔ | ✗ |
| 21110120 | XAC3730 | cheR | methyltransferase | VTNFpTPAVpTDILPLVESDIQRPISHIK(*) | ✗ | ✔ |
|  |  |  |  |  |  |  |
| **Posttranslational modification, protein turnover, chaperones** | | | | | | |
| 21106636 | XAC0542 | groEL | molecular chaperone GroEL | SALQNAApSIAGLMIpTpTEAMVADAPKK(*) | ✗ | ✔ |
| 21107041 | XAC0906 | ahpF | alkyl hydroperoxide reductase subunit F | LDpTNAAKR | ✔ | ✗ |
| 21107220 | XAC1080 | lon | ATP-dependent serine proteinase La | GNLILTGQLGNVMKESApSAALSVVR | ✗ | ✔ |
| 21107480 | XAC1321 | mucD | periplasmic protease | YVPFIQTDVAINQGNpSGGPLLNTR | ✗ | ✔ |
| 21107867 | XAC1675 | dsbE | C-type cytochrome biogenesis protein/thioredoxin | HIGVLpTPDAINDELLPAIApSLPK(*) | ✔ | ✔ |
| 21109029 | XAC2745 | - | metallopeptidase | MGTLVpTNLR | ✔ | ✔ |
| 21109079 | XAC2789 | - | peptidyl-prolyl cis-trans isomerase | TDSQpTFR | ✔ | ✗ |
| 21109127 | XAC2833 | - | extracellular serine protease | MNGpTSASAPTVAGVAALMLGANPQLTLR | ✔ | ✗ |
|  |  |  |  |  |  |  |
| **Inorganic ion transport and metabolism** | | | | | | |
| 21106360 | XAC0291 | oar | Oar protein | ANpTFTLVDTGGNpYVSVPLNNAELGMPAFK(*) | ✗ | ✔ |
| 21106547 | XAC0460 | phaD | monovalent cation/H+ antiporter subunit D | ApTTPQRR | ✗ | ✔ |
| 21106978 | XAC0849 | ssuA | sulfonate binding protein | QLpTELK | ✔ | ✗ |
| 21109027 | XAC2743 | oar | Oar protein | DTQHNQQWNPGApSSLPNIASSNLQDPTITGGR | ✔ | ✗ |
| 21109124 | XAC2830 | fhuA | TonB-dependent receptor | VQDVDQYELGLKSGpTpSAYDLYLTAFYNTFK(*) | ✗ | ✔ |
| 21109289 | XAC2980 | mgtE | Mg++ transporter | RRLIWLpSINLCTAFLASSVVSR | ✔ | ✗ |
| 21110440 | XAC4029 | catB | catalase precursor | QVAAVQAKDYSHLpTNDLVGAIKK | ✔ | ✗ |
| 21110713 | XAC4273 | - | OmpA-related protein | GQVAASQAGTTVTApTNLATGTTR | ✔ | ✗ |
|  |  |  |  |  |  |  |
| **Secondary metabolites biosynthesis, transport and catabolism** | | | | | | |
| 21108432 | XAC2197 | - | hemolysin-type calcium binding protein | SQpTLRFKSGVTAQDVVLR | ✔ | ✗ |
|  |  |  |  |  |  |  |
| **General function prediction only** | | | | | | |
| 21107225 | XAC1087 | gloB | hydroxyacylglutathione hydrolase | AASALpSpSEVDIFAELRR | ✗ | ✔ |
| 21109727 | XAC3375 | - | conserved hypothetical protein | AIDAAAQLLQQAGFKQGEILLVSDGADpSSAESSAR | ✗ | ✔ |
| 21110248 | XAC3847 | amaA | N-acyl-L-amino acid amidohydrolase | QTHGSAPWNGIDPIVApSADMIGAAQTVISRR | ✔ | ✗ |
|  |  |  |  |  |  |  |
| **Function unknown** | | | | | | |
| 21106334 | XAC0268 | - | conserved hypothetical protein | ALVNKIEPVpSWVLGDLLR | ✔ | ✗ |
| 21106608 | XAC0518 | - | conserved hypothetical protein | GAPQLpSGPQR | ✗ | ✔ |
| 21109403 | XAC3082 | - | conserved hypothetical protein | RFVpSKAVEQQIAQIK | ✔ | ✗ |
|  |  |  |  |  |  |  |
| **Signal transduction mechanisms** | | | | | | |
| 21107862 | XAC1671 | - | conserved hypothetical protein | DAHLHpSLCHGVISLER | ✗ | ✔ |
| 21108154 | XAC1938 | - | GGDEF family protein | SGMIGLLGpSWVLRQACR | ✗ | ✔ |
| 21108826 | XAC2555 | fixL | sensor histidine kinase | IpYSEVGEGSTVR | ✔ | ✔ |
| 21109423 | XAC3099 | pilJ | pilus biogenesis protein | NPAALSALEQpSQAQWTTMKK | ✔ | ✗ |
| 21110120 | XAC3730 | cheR | methyltransferase | VTNFpTPAVpTDILPLVESDIQRPISHIK(*) | ✗ | ✔ |
| 21110803 | XAC4358 | - | conserved hypothetical protein | KRLIGAVQDVpTDR | ✔ | ✔ |
|  |  |  |  |  |  |  |
| **Intracellular trafficking, secretion, and vesicular transport** | | | | | | |
| 21108666 | XAC2411 | acvB | virulence protein | HLQDSNpTAGHYGSVEVIRPEGTPK | ✗ | ✔ |
| 21109883 | XAC3515 | bcsC | cellulose synthase subunit C | ALAGSpTAAAQQpTPTGLADTLNAMAASGNGSR(*) | ✔ | ✗ |
|  |  |  |  |  |  |  |
| **No COG** | | | | | | |
| 21106133 | XAC0086 | - | hypothetical protein | IQRSTLpSR | ✔ | ✗ |
| 21106361 | XAC0292 | - | conserved hypothetical protein | pSKLTVITER | ✗ | ✔ |
| 21106542 | XAC0455 | - | conserved hypothetical protein | LLGLMSALLVLpTACNR | ✗ | ✔ |
| 21106702 | XAC0601 | - | hypothetical protein | IRSTPTFHEQTETGAPpSVAPLAR | ✗ | ✔ |
| 21107152 | XAC1013 | - | conserved hypothetical protein | RMpTAEEFDAWMKAR | ✗ | ✔ |
| 21107304 | XAC1162 | - | conserved hypothetical protein | VTKDGpTAR | ✗ | ✔ |
| 21107564 | XAC1397 | - | conserved hypothetical protein | IGSADpTGLR | ✗ | ✔ |
| 21107667 | XAC1491 | - | hypothetical protein | QPIpSAPMR | ✗ | ✔ |
| 21107745 | XAC1563 | - | hypothetical protein | pSDLAASLRPER | ✔ | ✗ |
| 21108386 | XAC2156 | - | conserved hypothetical protein | KGGQAVpSR | ✔ | ✗ |
| 21108419 | XAC2186 | - | conserved hypothetical protein | AGNIAQMLpTTGTEGAR | ✔ | ✗ |
| 21108495 | XAC2255 | - | hypothetical protein | VQQLQSAGTAMDVpYVVGSRGDDK | ✔ | ✗ |
| 21108700 | XAC2443 | - | conserved hypothetical protein | pYQDEIDGAAQDAAKGAGR | ✔ | ✗ |
| 21109156 | XAC2859 | - | hypothetical protein | ADQRVSQVVQDALPRLTQLSNQALTQpSLEPAVpTR | ✔ | ✗ |
| 21109232 | XAC2927 | hup | histone-like protein | AKpTAAK | ✗ | ✔ |
| 21109443 | XAC3118 | - | conserved hypothetical protein | GRVYTLANGQEWEQpTDTASLSAVRK | ✔ | ✗ |
| 21109917 | XAC3546 | xadA | outer membrane protein | AISLGGQpSR | ✗ | ✔ |
| 21109991 | XAC3612 | - | peptidase | pSADAGRGRK | ✔ | ✗ |
| 21110422 | XAC4012 | - | conserved hypothetical protein | TTPINAMRVATAGLDLLHTpTRLK | ✗ | ✔ |
| 21110646 | XAC4213 | - | shikimate kinase | DALEPLDVANVLNALpSK | ✗ | ✔ |
| 21110663 | XAC4228 | - | sialic acid-specific 9-O-acetylesterase | RGGLLGDAPpSR | ✔ | ✗ |
| 21110781 | XAC4338 | - | hypothetical protein | GITDGDSpSNDQTWMINLEK | ✗ | ✔ |

(a) and (b) Peptide was identified to be phosphorylated in rich medium (a) or plant (b). The tick indicates identified; the cross indicates not identified.

(*) Protein phosphorylated in multiple sites.
