# Supplementary material for: A Phosphorylation Switch on Lon Protease Regulates Bacterial Type III Secretion System in Host
Source: mBio. 2018 Jan 23;9(1):e02146-17. doi: 10.1128/mBio.02146-17 (PMC5784255; doi:10.1128/mBio.02146-17)
Supplement: TABLE S2 [file mbo001183690st2.docx]

|  | NB | | Plant | | Total | |
| --- | --- | --- | --- | --- | --- | --- |
| Individual functional categories | Peptides | *P*-value | Peptides. | *P*-value | Peptides. | *P*-value |
| J: Translation, ribosomal structure and biogenesis | 4 | 0.28 | 1 | 1.00 | 5 | 0.40 |
| K: Transcription | 2 | 1.00 | 1 | 0.72 | 3 | 0.63 |
| L: DNA Replication, recombination and repair | 2 | 1.00 | 0 | NA | 2 | 0.44 |
| D: Cell cycle control, cell division | 1 | 0.41 | 0 | NA | 1 | 0.58 |
| O: Posttranslational modification, protein turnover, chaperones | 5 | 0.05 | 5 | 0.02 | 8 | 0.02 |
| M: Cell wall/membrane/envelope biogenesis | 1 | 0.37 | 1 | 0.73 | 2 | 0.33 |
| P: Inorganic ion transport and metabolism | 5 | 0.19 | 3 | 0.45 | 8 | 0.07 |
| U: Intracellular trafficking, secretion, and vesicular transport | 1 | 1.00 | 1 | 1.00 | 2 | 1.00 |
| N: Cell motility | 3 | 0.22 | 1 | 1.00 | 4 | 0.33 |
| T: Signal transduction mechanisms | 3 | 1.00 | 5 | 0.18 | 6 | 0.65 |
| G: Carbohydrate transport and metabolism | 4 | 0.53 | 2 | 1.00 | 4 | 1.00 |
| E: Amino acid transport and metabolism | 2 | 1.00 | 1 | 0.72 | 2 | 0.33 |
| H: Coenzyme transport and metabolism | 4 | 0.12 | 1 | 1.00 | 5 | 0.22 |
| I: Lipid transport and metabolism | 2 | 0.69 | 2 | 0.39 | 4 | 0.36 |
| C: Energy production and conversion | 4 | 0.32 | 5 | 0.05 | 7 | 0.13 |
| Q: Secondary metabolites biosynthesis, transport and catabolism | 1 | 0.59 | 0 | NA | 1 | 1.00 |
| R: General function prediction only | 1 | 0.09 | 2 | 0.58 | 3 | 0.09 |
| S: Function unknown | 2 | 0.43 | 1 | 0.37 | 3 | 0.21 |
| No COG | 10 | 0.75 | 12 | 0.48 | 22 | 0.62 |

Table S2. Comparison of COG assignments with regard to phosphoproteins identified under different experimental conditions in *X. citri*

*P*-value is calculated using Fisher’s exact test. NA represents not available.
