# Supplementary material for: A Phosphorylation Switch on Lon Protease Regulates Bacterial Type III Secretion System in Host
Source: mBio. 2018 Jan 23;9(1):e02146-17. doi: 10.1128/mBio.02146-17 (PMC5784255; doi:10.1128/mBio.02146-17)
Supplement: TABLE S3 [file mbo001183690st3.docx]

| **Table S3. Strains and plasmids used in this study** | | |
| --- | --- | --- |
| Strain or plasmid | Relevant characteristic | Reference |
| Stains | | |
| *E. coli* | | |
| JM109 | *end*A1 *rec*A1 *gyr*A96 *thi* *hsd*R17 (r_k_−, m_k_+) *rel*A1 *sup*E44 ∆(*lac-pro*AB) [F’ *tra*D36 *pro*AB *laq*I^q^Z∆M15] | Promega |
|  |  |  |
| BL21(DE3) | *fhuA2 [lon] ompT gal (λ DE3) [dcm] ∆hsdS* | NEB |
| *Xanthomonas* spp. | |  |
| 306 | *X. citri* subsp. *citri* wild type strain, Rif^r^ | ^1^ |
| *∆lon* | *lon* deletion mutant of strain 306 | This study |
| *∆lon* OE | *∆lon* carrying pTF53-Lon for over-expression | This study |
| *∆lon* (*lon*) | *∆lon* carrying *lon* full length in its chromosome with its native promoter | This study |
| Plasmids |  |  |
| pNTPS138 | Suicide vector for generation of gene knockouts, *sacB* and Km^r^ | This study |
| pPM7g | Integrative plasmid containing a fragment *amy*106–912, Km^r^ | ^2^ |
| pTF53 | pUFR053 derivative containing the *trp* promoter and a 3 × Flag epitope-encoding sequence at carboxyl terminus, Gm^r^ | ^3^ |
| pNP138-Lon | pNPTS138 derivative for generation of *∆lon* | This study |
| pPM7g-Lon | pPM7g derivative encoding Lon under control of its native promoter | This study |
| pPM7g-P_hrpB1_::GUS | pPM7g derivative encoding GUS under control of HrpB1 promoter | This study |
| pPM7g-P_hrcU_::GUS | pPM7g derivative encoding GUS under control of HrcU promoter | This study |
| pPM7g-P_hrcQ_::GUS | pPM7g derivative encoding GUS under control of HrcQ promoter | This study |
| pTF53-HrpX | pTF53 derivative encoding HrpX-Flag | This study |
| pTF53-Lon | pTF53 derivative encoding Lon-Flag | This study |
| pTF53-LonS654A | pTF53 derivative encoding LonS654A-Flag | This study |
| pTF53-LonS654E | pTF53 derivative encoding LonS654E-Flag | This study |
| pTF53-LonS654D | pTF53 derivative encoding LonS654D-Flag | This study |
| pBGT | pBBR1MCS-5 derivative containing the *trp* promoter, Gm^r^ | This study |
| pBKT | pBBR1MCS-2 derivative containing the *trp* promoter, Km^r^ | This study |
| pBGT-HrpG6his | pBGT derivative encoding HrpG6his | This study |
| pBKT-HrpG6his | pBKT derivative encoding HrpG6his | This study |
| pBGT-HrpG6hisΔN20 | pBGT derivative encoding HrpG6hisΔN20 | This study |
| pBGT-6hisHrpG | pBGT derivative encoding 6hisHrpG | This study |
| pBRG-GFP | pBBR1MCS-5 derivative encoding GFP | This study |
| pBRG-GFP_1-15_ | pBBR1MCS-5 derivative encoding GFP_1-15_ | This study |
| pBRG-GFP_1-20_ | pBBR1MCS-5 derivative encoding GFP_1-20_ | This study |
| pBRG-GFP_1-25_ | pBBR1MCS-5 derivative encoding GFP_1-25_ | This study |
| pBRG-GFP_1-30_ | pBBR1MCS-5 derivative encoding GFP_1-30_ | This study |
| pBRG-GFP_1-50_ | pBBR1MCS-5 derivative encoding GFP_1-50_ | This study |
| pMAL-c5X | Cytoplasmic expression of MBP-fusion protein, Ap^r^ | NEB |
| pMAL-Lon | pMAL-c5X derivative encoding MBP-Lon | This study |
| pBbE1a-RFP | Expression vector encoding RFP, Ap^r^ | Addgene |
| pBbE1a-HrpG_1-15_::RFP | pBbE1a-RFP derivative encoding GFP_1-15_ | This study |
| pBbE1a-HrpG_1-50_::RFP | pBbE1a-RFP derivative encoding GFP_1-50_ | This study |
| pBbE1a-Lon | pBbE1a-RFP derivative encoding wild type Lon-6his | This study |
| pBbE1a-LonS654A | pBbE1a-RFP derivative encoding LonS654A-6his | This study |
| pBbE1a-LonS654E | pBbE1a-RFP derivative encoding LonS654E-6his | This study |

Rif^r^, Km^r^, Amp^r^, Gm^r^ indicate resistance to rifampicin, kanamycin, ampicillin, and gentamicin, respectively.

Reference:

1. da Silva, A. C. R. *et al.* Comparison of the genomes of two *Xanthomonas* pathogens with differing host specificities. *Nature* **417,** 459–63 (2002).

2. Martins, P. M. M. *et al.* Subcellular localization of proteins labeled with GFP in *Xanthomonas* *citri* ssp. *citri*: targeting the division septum. *FEMS Microbiol. Lett.* **310,** 76–83 (2010).

3. Zhou, X., Hu, X., Li, J. & Wang, N. A Novel Periplasmic Protein, VrpA, Contributes to Efficient Protein Secretion by the Type III Secretion System in *Xanthomonas* spp. *Mol. Plant. Microbe. Interact.* **28,** 143–53 (2015).
