# Supplementary material for: A Phosphorylation Switch on Lon Protease Regulates Bacterial Type III Secretion System in Host
Source: mBio. 2018 Jan 23;9(1):e02146-17. doi: 10.1128/mBio.02146-17 (PMC5784255; doi:10.1128/mBio.02146-17)
Supplement: TABLE S4 [file mbo001183690st4.docx]

| **Table S4. Primers used in this study.** | |
| --- | --- |
| Primer name | Sequences (5’ to 3’) (Underline represents restriction sites) |
| *For inserted complementation* | |
| LonPro-F | AAATCTAGACGCGTGGCCTGCGCACCATCGT |
| Lon-Rflag | GATCTTTATAATCACCGTCATGGTCTTTGTAGTCGTGCTTGACGCGGGTACCGGG |
| flagXhoI-R | AAACTCGAGTTACTTGTCATCGTCATCCTTGTA |
| *For over-expression of Lon* | |
| LonInF-F | AGGAAAAACATATGGGTACCATGGCCCAGTCCCAACCAGAAG |
| LonInF-R | AGGAAGGATCCTGCGGTACCGTGCTTGACGCGGGTACCGGGC |
| *For over-expression of HrpG, HrpX and HrpG derivatives* | |
| HrpX-InF | AGGAAAAACATATGGGTACCATGATCCTTTCCACTTACTTTG |
| HrpX-InR | AGGAAGGATCCTGCGGTACCCCGCTGCAAGGTCTCCATCGGC |
| HishrpG-F | AAAGGTACCATGCATCACCATCACCATCACAACGACCACTCTCCCCCCAACG |
| HishrpG-R | ATACTCGAGTCAGCAGGCGGCTGCGTGATGTG |
| hprGC20-R | ATACTCGAGTGCCTCCGTATCGTGCAGCGCG |
| N20hprG-F | AAAGGTACCATGCTGGTCTCGCAGGTCAACGCCAG |
| N20hprG-R | ATACTCGAGTCAGTGATGGTGATGGTGATGGCAGGCGGCTGCGTGATGTGCAG |
| *For GUS reporter assay* |  |
| HrcQpro-F | AAATCTAGAGCCACCGAGTGGATACGCACGC |
| HrcQGUS-R | GGGTTTCTACAGGACGTAACATCCTGCTCAGCTGTGCGCGCTCG |
| HrcQGUS-F | CGAGCGCGCACAGCTGAGCAGGATGTTACGTCCTGTAGAAACCC |
| HrcUpro-F | AAATCTAGAGCGGCCAGGACCGCGGCAGCGT |
| HrcUGUS-R | GGGTTTCTACAGGACGTAACATTGCCCTCTCCTTCCGCGATCGC |
| HrcUGUS-F | GCGATCGCGGAAGGAGAGGGCAATGTTACGTCCTGTAGAAACCC |
| HrpB1pro-F | AAATCTAGAGCGCGGTTTATTGCCGTTGCAT |
| HrpB1GUS-R | GGGTTTCTACAGGACGTAACATACTGCACCTCGTTTTAATGACA |
| HrpB1GUS-F | TGTCATTAAAACGAGGTGCAGTATGTTACGTCCTGTAGAAACCC |
| GUS-R | AAACTCGAGTCATTGTTTGCCTCCCTGCTGC |
| *For site-direct mutagenesis* | |
| LonS654E-F | GGAATCGGCAGAGGCTGCATTG |
| LonS654E-R | TTCATGACATTGCCGAGC |
| LonS654D-F | GGAATCGGCAGATGCTGCATTGTC |
| LonS654D-R | TTCATGACATTGCCGAGC |
| LonS654A-F | GGAATCGGCAGCCGCTGCATTGTC |
| LonS654A-R | TTCATGACATTGCCGAGC |
| *For QRT-PCR* |  |
| LOBqPCR-F | CACGCTTCCCTTCTCCTAATC |
| LOBqPCR-R | CAGCACAAGGGCTAAGAACTA |
| qGAPDH-F | GGGCGTCAACGAGAAAGAATA |
| qGAPDH-R | TGGAATGGACAGTGGTCATAAG |
| qgyrB-F | ATGACCGACGAACAAAACACCC |
| qgyrB-R | CGCCGACACGCCTTCTTCCTTG |
| hrpGqrt-F | GATCTTCGATGCCAGCTATGT |
| hrpGqrt-R | CGATACCAGGCCAGAATGTT |
| hrpXqrt-F | CGAACAAGCGTTACTGCTCTA |
| hrpXqrt-R | GAGATATCGTCGCTGACGTG |
| hrcQqrt-F | CATGCAGCACGATACGTTTG |
| hrcQqrt-R | AAACCCGACAACGTCGATAG |
| hrcUqrt-F | TCCAGGGATTGGTGTTGATG |
| hrcUqrt-R | GGGATTGAGGGAATCGAACTT |
| hrpB1qrt-F | GGACTCACCCATGACAAGATTC |
| hrpB1qrt-R | TACCGCGCTTGATGGAAATC |
| *For HrpG N-terminal-GFP derivatives* | |
| GFPF | AAAGGTACCATGAGTAAAGGAGAAGAACTTT |
| GFPR | ATACTCGAGCTATTTGTATAGTTCATCCATG |
| G50GFPF | ATGAGTAAAGGAGAAGAACTTTTCACTG |
| G50GFPR | CCGCAGCGTACGCAAAAG |
| G30GFPF | ATGAGTAAAGGAGAAGAACTTTTCACTGG |
| G30GFPR | TGCGAGGCTGGCGTTGAC |
| G25GFPR | GACCTGCGAGACCAGGCG |
| G20GFPF | ATGAGTAAAGGAGAAGAACTTTTCACTGGAG |
| G20GFPR | GCGCGCATCCTGCGTCAA |
| G15GFPF | ATGAGTAAAGGAGAAGAACTTTTC |
| G15GFPR | CAACAGGAACACCGATCC |
| hrpG80GFPF | AAAGGTACCATGAACGACCACTCTCCCCCCA |
| hrpG80GFPR | AAAAGTTCTTCTCCTTTACTCATGCCGCTATGGCAGCGCTGCCATG |
| GFPhrpG80F | CATGGCAGCGCTGCCATAGCGGCATGAGTAAAGGAGAAGAACTTTT |
| *For protein expression and purification* | |
| MBP-LonF | AAAGTCGACATGGCCCAGTCCCAACCAGAAG |
| MBP-LonR | ATAGAATTCGTGCTTGACGCGGGTACCGGGC |
| RFPC6hisF | CACCACCACTAAGGATCCAAACTCGAGTAAGGATC |
| RFPC6hisR | ATGATGATGAGCACCGGTGGAGTGACG |
| HrgRFP1a-F | AAACATATGAACGACCACTCTCCCCCCAACGC |
| HrgRFP1a-R | AAAGGATCCTTAGTGGTGGTGATGATGATGAGCACCGGTGGAGTGACGAC |
| 1a50RFPm-F | ATGGCGAGTAGCGAAGAC |
| 1a50RFPm-R | CCGCAGCGTACGCAAAAG |
| 1a15RFPm-R | CAACAGGAACACCGATCC |
| Lon1a-F | AAACATATGGCCCAGTCCCAACCAGAAGTTC |
| Lon1a-R | AAAGGATCCTTAGTGGTGGTGATGATGATGGTGCTTGACGCGGGTACCGG |
| 1a50RFPm-F | ATGGCGAGTAGCGAAGAC |
| 1a50RFPm-R | CCGCAGCGTACGCAAAAG |
|  |  |
|  |  |
|  |  |
